# One‐Step Synthesis of Closed‐Loop Recyclable and Thermally Superinsulating Polyhexahydrotriazine Aerogels

**DOI:** 10.1002/adma.202412502

**Published:** 2024-11-04

**Authors:** Chang‐lin Wang, Yi‐Ru Chen, Fabian Eisenreich, Željko Tomović

**Affiliations:** ^1^ Polymer Performance Materials Group Department of Chemical Engineering and Chemistry and Institute for Complex Molecular Systems (ICMS) Eindhoven University of Technology Eindhoven 5600 MB The Netherlands

**Keywords:** aerogels, hexahydrotriazine, recycling, sustainability, thermal insulation, thermal stability

## Abstract

Organic aerogels are an advanced class of materials renowned for their ultralow thermal conductivity and highly porous architecture, making them ideal for applications in thermal insulation, catalysis, and chemical absorption. However, these polymeric networks pose environmental concerns as their permanently crosslinked scaffold makes recycling back to the original monomers virtually impossible. To tackle this issue and develop next‐generation organic aerogel, a set of polyhexahydrotriazine (PHT) aerogels specifically designed for closed‐loop chemical recycling are prepared. Remarkably, these innovative materials can selectively be synthesized in a one‐step condensation reaction using commercially available aromatic amines. They showcase outstanding thermally insulating performance, along with strong mechanical performance, pronounced thermal stability, and intrinsic hydrophobicity, all achieved without the need for additional modifications. More importantly, these aerogels exhibit quantitative depolymerization under acidic aqueous conditions, achieving high yields and purities of the recovered monomers. The successful preparation of fresh organic aerogels from recycled monomers with nearly identical material properties underscores the efficiency and reliability of this recycling process. The facile one‐step synthesis process, combined with the high‐performance properties and excellent recyclability of these PHT aerogels, accelerates the advancement of sustainable thermally superinsulating materials.

## Graphical abstract

The simple one‐step synthesis of closed‐loop recyclable PHT aerogels is presented, which are made from commercially available aromatic amines and PFA. These PHT aerogels display thermal superinsulation, pronounced thermal stability, great mechanical performance, and intrinsic hydrophobicity. Furthermore, PHT aerogels can be depolymerized under acidic aqueous conditions, allowing the monomers to be reused to prepare fresh aerogels.

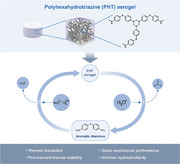

## 1 Introduction

The ever‐increasing consumption of energy has surged to its highest levels so far, with projections indicating a 14% growth by the year 2050.^[^ ^1^ ^]^ Notably, the heating and cooling of residential sectors account for ≈48% of the global building energy consumption.^[^ ^2^, ^3^ ^]^ Hence, effective thermal insulation techniques are aspired to curb the continuous rise in energy consumption. Aerogels, listed among the top ten emerging technologies of chemistry by IUPAC, are considered suitable solutions for effective energy preservation.^[^ ^4^ ^]^ Organic aerogels are equipped with unique material properties, such as low density (<0.2 gcm^−3^), large specific surface area (>100 m^2^g^−1^), high porosity (>80%), and ultralow thermal conductivity (<20 mWm^−1^K^−1^).^[^ ^5^, ^6^ ^]^ Due to these attributes, they outperform existing commercial products, such as polystyrene foams, polyurethane foams, and glass/rock wools, in terms of their thermally superinsulating performance.^[^ ^7^, ^8^ ^]^ Organic aerogels, typically composed of crosslinked polymers, offer high versatility in their molecular design, which are developed for different applications, such as chemical absorption,^[^ ^9^ ^]^ catalysis,^[^ ^10^ ^]^ and energy storage.^[^ ^11^, ^12^ ^]^ Various functional moieties have been introduced to construct organic aerogels, such as polyamide,^[^ ^13^, ^14^ ^]^ polybenzoxazine,^[^ ^15^, ^16^ ^]^ polyimide,^[^ ^17^, ^18^ ^]^ or polybenzimidazole aerogels.^[^ ^19^, ^20^ ^]^ These materials exhibit strong mechanical performance, exceptional thermal stability, and superior thermal insulation properties, making them stand out as high‐performance aerogel materials. However, the robust covalent bonds forming these polymeric networks are considered irreversible and non‐recyclable, leading to environmental concerns regarding their end‐of‐life disposal. Consequently, creating aerogel materials with exceptional properties, along with facile synthetic protocols and efficient recyclability, represents a formidable challenge.

A promising strategy to achieve the recyclability of organic aerogels lies in introducing reversible chemical bonds, such as imines, acetals, or diketoenamines in the polymeric scaffold.^[^ ^21^ ^]^ These bonds can be selectively cleaved under specific conditions, which allows the retrieval of the original monomers and the subsequent preparation of fresh organic aerogels.^[^ ^22^, ^23^, ^24^, ^25^ ^]^ However, a comprehensive molecular design is required to bestow aerogels with high‐performance properties, such as improved mechanical properties,^[^ ^17^, ^26^, ^27^ ^]^ enhanced thermal/flame resistance,^[^ ^20^, ^24^, ^28^ ^]^ and additional hydrophobicity.^[^ ^25^, ^29^, ^30^ ^]^ Typical approaches often involve additional chemical modifications of the monomers or the aerogels post‐synthetically.^[^ ^31^, ^32^ ^]^ These intricate and extensive design strategies may limit the accessibility and scalability of aerogel production. Therefore, there is a pressing need for a simplified synthetic protocol to overcome these challenges and fully unleash the potential of aerogels as innovative, sustainable thermally superinsulating materials.

**Figure 1:**
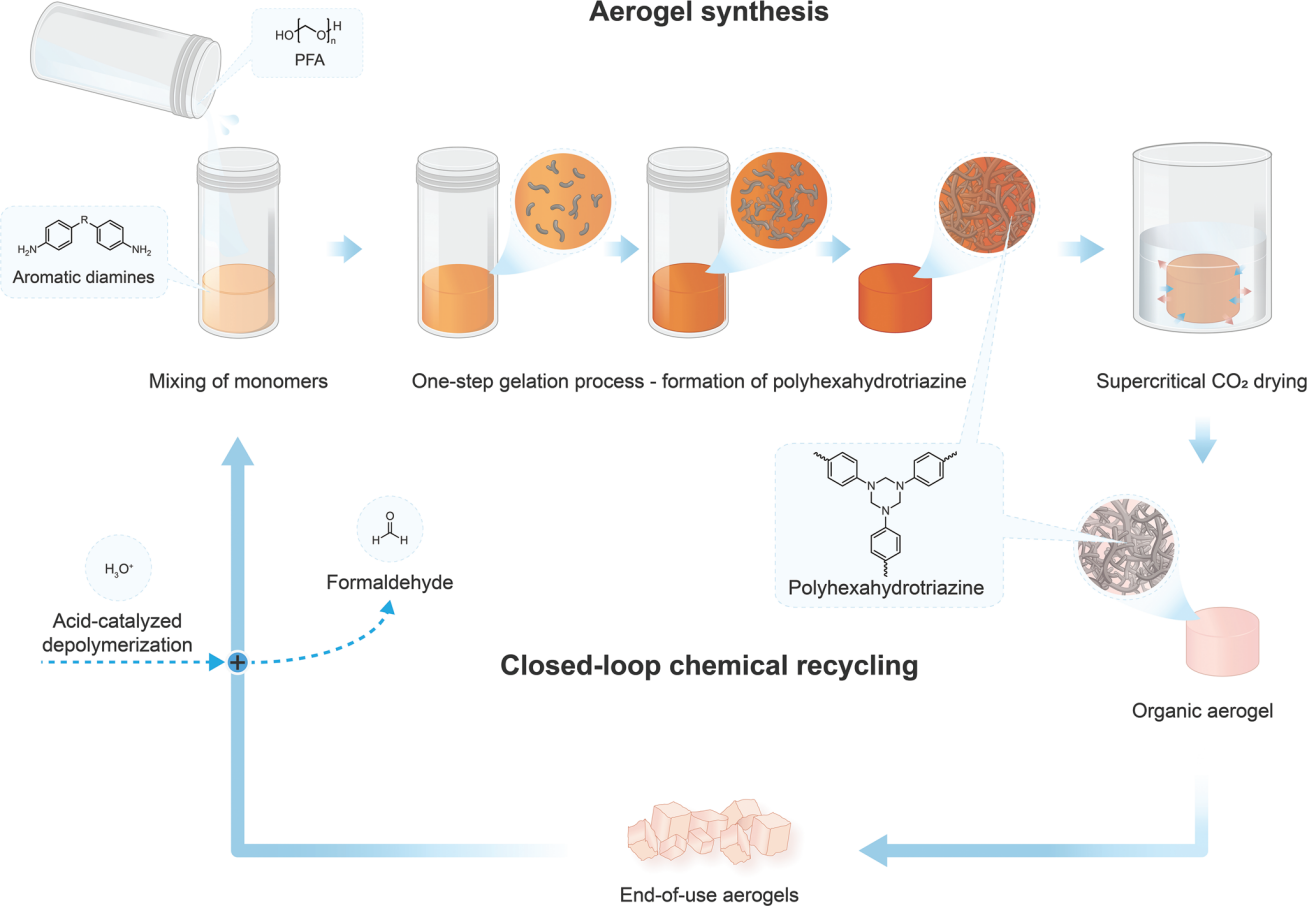
Circular flowchart of PHT aerogel synthesis and the closed‐loop chemical recycling scheme.

In this work, we utilize hexahydrotriazines (HTs) as robust and chemically reversible crosslinking motifs to develop PHT organic aerogels, specifically designed for efficient closed‐loop recycling (**Figure** **1**).^[^ ^33^ ^]^ Prepared from the condensation between multifunctional amines and formaldehyde, PHTs are renowned for the reversible nature of the HT bonds, which are widely applied in the development of various recyclable polymer materials.^[^ ^33^, ^34^, ^35^, ^36^, ^37^, ^38^, ^39^, ^40^, ^41^, ^42^ ^]^ Since both building blocks are commercially available and the synthesis only requires one simple step, the fabrication of PHT aerogel is considered more straightforward and facile than other recyclable aerogels in literature.^[^ ^33^, ^42^ ^]^ For a successful synthesis, it is essential to exclude the presence of unwanted hemiaminal (HA) intermediate structures, which could lower the thermal resistance and mechanical performance of the final materials.^[^ ^33^, ^42^ ^]^ Typically, these intermediates form when formaldehyde is used in excess and require post‐curing procedures to ensure the full trimerization into HT structures. Our developed protocol effectively avoids both issues. As a result, the prepared PHT aerogels show low bulk density (<0.2 gcm^−3^), high porosity (>82%), and large specific surface area (≈200 m^2^g^−1^). They also attain ultralow thermal conductivity with the value of 0.019 Wm^−1^K^−1^, serving as high‐performance thermally superinsulating materials. Moreover, closed‐loop recycling of our PHT aerogels was achieved by applying acidic aqueous conditions to induce the selective cleavage of methylene moieties within the PHT network. The high‐yielding recovery (up to 90%) of pure amine monomers allows for the preparation of fresh aerogels with nearly identical properties to the original ones. Additionally, the outstanding characteristics of PHT materials extend beyond recyclability, featuring great thermal resistance (T*_d5%_* > 330 °C), improved mechanical properties (ɛ > 1.4MPa), and intrinsic hydrophobicity without prior/post‐modifications. These aerogels represent a new generation of circular thermally insulating materials that can be prepared in a simple and straightforward manner, while featuring outstanding thermal and mechanical performance.

## 2 Results and Discussion

### 2.1 Design and Fabrication of PHT Aerogels

**Figure 2:**
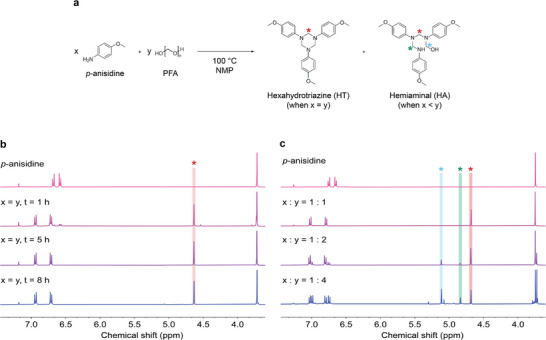
a) Reaction scheme of model compound *p*‐anisidine reacting with different molar ratios of PFA. b) ^1^H NMR spectra (400 MHz, 25 °C, CDCl_3_) of the reaction between *p*‐anisidine and one molar equivalent of PFA monitored at different points in time. c) ^1^H NMR spectra (400 MHz, 25 °C, CDCl_3_) of the reaction between *p*‐anisidine and different molar ratios of PFA (1, 2, and 4 eq.) after 8 h.

The preparation of the PHT network involves the trimerization reaction between aromatic amines and paraformaldehyde (PFA).^[^ ^42^, ^43^ ^]^ To gain a fundamental understanding of its mechanism, model studies based on *p*‐anisidine as an aromatic amine and PFA were conducted. *p*‐Anisidine was reacted with an equimolar amount of PFA relative to the amine functional groups in *N*‐methylpyrrolidone (NMP) at 100 °C for 8 h (**Figure** **2**a). Aliquots were taken at various time points during the reaction and analyzed using NMR spectroscopy. New signals indicating the formation of HT appeared at 4.8 ppm in the ^1^H NMR spectra and at 72 ppm in the ^13^C NMR spectra, which was completed within 5 h (Figure 2b; Figure S1a, Supporting Information). To elucidate the role of PFA in HT formation, *p*‐anisidine was reacted with PFA in varying molar equivalents. While the equimolar ratio of PFA to aromatic amine exclusively produced the desired HT structure, higher ratios of PFA led to the formation of undesired HA intermediates. These were characterized by a distinct chemical shift at 90 ppm in the ^13^C NMR spectra, indicating the presence of a hydroxymethylene moiety (Figure 2c; Figure S2b, Supporting Information). Hence, the exclusive formation of HT structures without HA byproducts was achieved using our optimized one‐step synthesis protocol.

**Figure 3:**
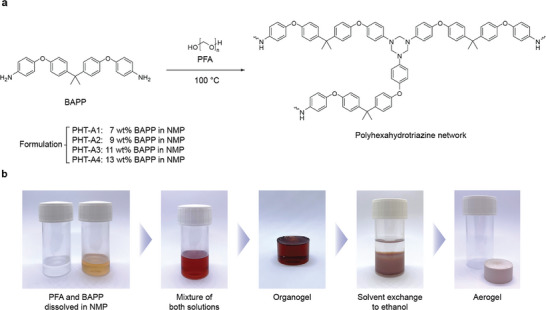
a) Reaction scheme illustrating the polycondensation reaction of BAPP with PFA to form a crosslinked PHT network. b) Schematic representation of the aerogel synthesis protocol. Precursors were dissolved separately in NMP, mixed at 100 °C for 8 h, and cured overnight at room temperature to obtain stable organogels. The NMP solvent was exchanged twice, for 24 h each time, with 0.1 M NaOH, water, and ethanol before supercritical drying with CO_2_ was applied to prepare PHT aerogels.

After gaining insights into the HT formation through the one‐step synthesis, we employed the same reaction conditions using six commercially available multifunctional amines to identify the most effective amine precursor for aerogel synthesis (Scheme S1, Supporting Information). To form the initial organogel, different organic solvents, such as NMP, *N,N*‐dimethylformamide (DMF), *N,N*‐dimethylacetamide (DMAc), and dimethyl sulfoxide (DMSO), were examined (Table S1, Supporting Information). First, the amine monomers and PFA were dissolved separately in the organic solvent at 100 °C. The amount of PFA was kept at an equivalent molar ratio to the amine functional groups of the amine precursor. The trimerization reaction was initiated by combining the solutions at the same temperature, resulting in the formation of a stable organogel after 8 h. The solvent of the organogel was subsequently exchanged twice, for 24 h each time, using a 0.1 m sodium hydroxide solution, water, and ethanol to completely remove the residue organic solvent. Afterward, supercritical CO_2_ drying was conducted to remove the solvent from the ethanol‐saturated organogels without disrupting their delicate polymer network structure (**Figure** **3**a−b). The material properties of the produced organic aerogels were then thoroughly analyzed (Table S2, Supporting Information). Among the various combinations tested, using 2,2‐bis[4‐(4‐aminophenoxy)phenyl]propane (BAPP) as the amine and NMP as the organic solvent yielded the aerogel material with the best performance, as indicated by its low bulk density and high specific surface area. Thus, BAPP was chosen as the ideal amine for optimizing the characteristics of PHT aerogels by investigating the correlation between initial amine concentration (in the range of 7–13 wt.%) and material properties of the final aerogels (Scheme S1, Supporting Information). The aerogel samples are labeled accordingly as PHT‐A1 to PHT‐A4 throughout this study (Figure 3a).

**Figure 4:**
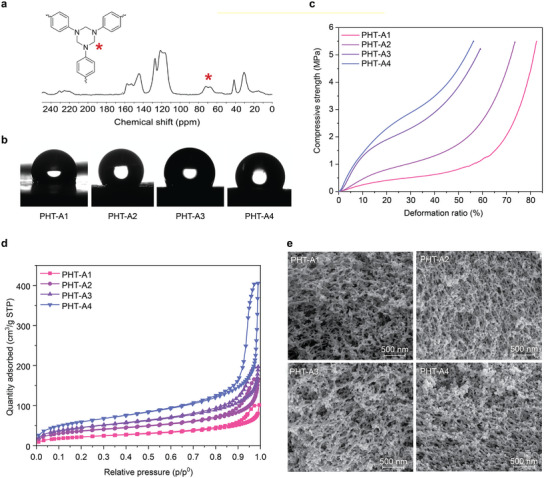
a) Solid‐state ^13^C MAS NMR spectra of PHT‐A2. b) Water contact angle images of PHT aerogels. c)Stress‐deformation curves of PHT aerogels. d) Nitrogen physisorption isotherms of PHT aerogels. e) SEM images of PHT aerogels.

Before discussing the results of the optimization study, we first confirmed the formation of PHT within our aerogels by using ^13^C MAS NMR spectroscopy. **Figure** **4**a shows the ^13^C MAS NMR spectra of PHT‐A2, where the chemical shift at 72 ppm validates the methylene carbon of the HT structure. Importantly, the absence of a chemical shift at 90 ppm, which corresponds to the intermediate HA structure, verifies the HT formation was quantitative without any side product formation. To further investigate this observation, two different PHT aerogels, PHT‐O1 and PHT‐O2, were prepared based on different molar ratios between ODA and PFA (Figure S3, Supporting Information). According to their ^13^C MAS NMR spectra, the chemical shift belongs to the HA structure at 90 mm from PHT‐O2 aerogels, confirming the HA formation within the PHT network while preparing them using the higher molar amount of PFA relative to the amine functional groups.

### 2.2 Physical and Microstructural Properties of PHT Aerogels

The physical properties of PHT aerogels, including bulk density, linear shrinkage, and porosity, are summarized in **Table** **1**. All samples exhibited linear shrinkage (i.e., diameter change) of less than 20%, resulting in the low bulk density of the PHT aerogels. Specifically, PHT‐A1 to A3 had a bulk density of less than 0.2 gcm^−3^, confirming their lightweight nature. The porosity of the PHT aerogels, measured using a helium pycnometer, revealed highly porous networks with porosity values up to 92%, indicating air‐filled scaffolds. For long‐term applications, it is crucial to test the water resistance of aerogels as water absorption can significantly reduce their thermally superinsulating capabilities. To assess this, we performed a water uptake test by submerging the aerogel samples in deionized water for 24 h. The weight difference before and after submersion was recorded, and all PHT aerogel samples showed less than 5% water uptake, suggesting their intrinsic water repellence (Table 1). Additionally, their water affinity was measured using a water contact angle test. Figure 4b demonstrates that all aerogels exhibited high water contact angle values of at least 90°. This indicates that PHT aerogels possess great intrinsic hydrophobicity, making them ideal for thermally superinsulating applications.

**Table 1:** General material properties of PHT aerogels.

Name	Bulk density ρ_b_ [mgcm^−3^]	Linear shrinkage [%]^a)^	Skeletal density ρ_s_ [gcm^−3^]	Porosity *Π* [%]^b)^	Contact angle [°]	Water uptake [%]^c)^	Young's modulus [MPa]^d)^	Compressive strength [MPa]^e)^
PHT‐A1	98	14	1.22	92	110 ± 8	0.8	0.99 ± 0.55	0.25 ± 0.02
PHT‐A2	145	17	1.22	88	130 ± 4	2.8	1.28 ± 0.12	0.45 ± 0.02
PHT‐A3	189	18	1.19	87	125 ± 2	1.0	1.72 ± 0.38	1.28 ± 0.24
PHT‐A4	212	16	1.21	82	98 ± 6	1.1	4.37 ± 2.28	1.46 ± 0.33

^a)^ Linear shrinkage was calculated based on the diameter change of the sample;

^b)^ Porosity was calculated via equation: *Π* = (1‐ρ_b_/ρ_s_) × 100%;

^c)^ Water uptake ratios were determined by the samples’ weight difference before and after submerging in distilled water for 24 h (sample size: 25 mm diameter and 15 mm height);

^d)^ Compressive modulus was calculated from the stress‐deformation curve obtained using a sample of 25 mm diameter and 15 mm height;

^e)^ Compressive strength at 10% deformation ratio.

As PHTs are generally known for their strong mechanical performance,^[^ ^40^, ^43^ ^]^ the mechanical properties of our PHT aerogels were characterized by uniaxial compression tests. The stress‐deformation curves under compressive testing showcase the excellent mechanical performance of PHT aerogels (Figure 4c). The compressive modulus of these aerogels ranges from 1.0 to 4.5 MPa, surpassing other organic aerogel materials (Figure S4, Supporting Information). Additionally, at a deformation ratio of 10%, the compressive strength of PHT aerogels varied between 0.4 and 1.6 MPa, indicating their ability to withstand high stress. Furthermore, all PHT aerogels tolerated high compressive strain without observable blisters or cracks up to a deformation of 80%, confirming their robust mechanical nature (Figure S5, Supporting Information). Notably, an increase in aerogel bulk density was associated with an improvement in compressive modulus.

The microstructural and morphological features of PHT aerogels were further characterized using nitrogen sorption porosimetry. According to the physisorption isotherms, all PHT aerogels display Type IV characteristics as per the IUPAC classification. The hysteresis observed in the desorption isotherm around the region p/p^0^ > 0.7 suggests a wide range of mesopore sizes in all samples. Additionally, PHT aerogels exhibit high specific surface areas ranging from 78 to 215 m^2^ g^−1^ (**Table** **2**). There is also a trend of higher adsorbed gas volumes with increasing bulk density (Figure 4d). Among them, PHT‐A4 shows a predominantly higher specific surface area and larger pore volume. To further investigate these correlations and study their overall surface morphologies, PHT aerogels were characterized using scanning electron microscopy (SEM, Figure 4e). Similar branched‐like structures were observed in the cross‐sections of all PHT aerogels. We measured the skeleton widths, indicating that all polymer skeleton dimensions fall within the range of 20–50 nm (Figure S6a, Supporting Information; Table 2). This suggests that the variations in gelling concentration do not influence the overall polymer skeleton formation. The differences in specific surface area and pore volume, however, can be attributed to the increased initial concentration of the feedstock molecules, where the formation of a denser polymer network facilitates the creation of smaller complementary mesopores.

**Table 2:** Microstructural and thermal properties of PHT aerogels.

Name	Specific surface area [m^2^g^−1^]	Pore volume [cm^3^g^−1^]	Skeleton width [nm]^a)^	Thermal conductivity [mWm^−1^K^−1^]^b)^	Decomposition temperature at 5% weight loss *T*_d5%_ [°C]^c)^	Char yield at 793 °C [%]^c)^
PHT‐A1	78	0.16	36 ± 10	20.1 ± 4.5E−02	336	18.5
PHT‐A2	129	0.25	30 ± 6	18.8 ± 1.9E−02	336	16.6
PHT‐A3	162	0.30	30 ± 7	20.9 ± 2.9E−02	340	18.2
PHT‐A4	215	0.64	34 ± 6	23.3 ± 9.0E−02	326	15.9

^a)^ Measured using ImageJ software, 100 data points were taken;

^b)^ Measured with a heat flow meter (Thermtest Inc., HFM‐25);

^c)^ Measured by TGA.

### 2.3 Thermal Properties of PHT Aerogels

**Figure 5:**
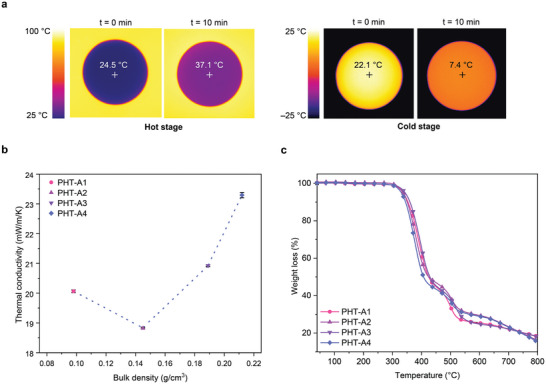
a) Top‐view IR images of a PHT‐A2 aerogel specimen (10 mm thickness) placed on a hot (100 °C) and a cold stage (−25 °C) at the beginning and after 10 min. b) Thermal conductivity of PHT aerogels in relation to bulk density. c) TGA measurements of PHT aerogels from 40 to 793 °C with a ramp rate of 10 °C min^−1^.

Owing to their nanoscale architectures and the related Knudsen effect, PHT aerogels are anticipated to demonstrate exceptional thermal insulation performance.^[^ ^44^ ^]^ The thermal conductivities of the PHT aerogels were measured using a heat flow meter, following the ASTM C518 standard (Table 2). With thermal conductivities below 23.3 mW m^−1^K^−1^, PHT aerogels demonstrate comparable or superior performance to other aerogel materials (Figure S7, Supporting Information). Among them, PHT‐A2 shows the lowest thermal conductivity with a value of 18.8 mW m^−1^K^−1^. To visualize its thermal insulation property, we used an IR camera to capture the insulation behavior of PHT‐A2 (**Figure** **5**a). PHT‐A2 with 10 mm thickness was placed onto a heating/cooling stage and the temperature was set to 100 and −25 °C, respectively. After 10 min, the middle point temperatures on the surface of the aerogels were recorded. The low thermal conductivity of PHT‐A2 restricts the heat transfer through the specimen, causing only moderate changes in its surface temperature compared to the stage. To gain a better understanding, we plotted the thermal conductivity values of PHT aerogels against the bulk densities (Figure 5b). Interestingly, PHT‐A3 and PHT‐A4 exhibit slightly higher thermal conductivities compared to PHT‐A2, despite their high surface areas. This can be attributed to their increased bulk density, which leads to higher solid conduction and partially offsets the benefits of the Knudsen effect. Conversely, PHT‐A1, with its lower density, shows higher thermal conductivity values compared to PHT‐A2, suggesting that factors beyond density, such as mesopore volume and pore size distribution, also play critical roles in determining thermal insulation performance.^[^ ^45^, ^46^ ^]^ In summary, the minimum thermal conductivity value for PHT aerogels is found to be 0.019 Wm^−1^K^−1^ for PHT‐A2, which benefits from its mesoporous network and reduced bulk density.

Since our PHT aerogels consist of a high content of aromatic and HT moieties, we can expect pronounced thermal resistance from these materials. We investigated the thermal stability of PHT aerogels using thermogravimetric analysis (TGA, Figure 5c). The high *T*_d5%_ values above 300 °C can be originated from the primary HT structures present in PHT aerogels. These results also validate the full formation of the HT structure without the presence of unwanted intermediates, specifically HAs. It has been found that HAs have a significantly lower decomposition temperature of 200 °C, which can greatly reduce the thermal stability of PHT aerogels.^[^ ^33^ ^]^ Therefore, the absence of HAs in PHT aerogels plays a crucial role in maintaining their exceptional thermal stability. Furthermore, the long‐term thermal stability of PHT aerogels when subjected to temperatures of 80, 100, and 120 °C for 3 h, respectively, was tested. The weight loss of the sample was less than 0.02% under all temperatures (Figure S8, Supporting Information), indicating no degradation of the aerogel networks during the long‐term heating process. These results demonstrate the excellent thermal stability of PHT aerogels.

### 2.4 Closed‐Loop Recycling of PHT Aerogels

**Figure 6:**
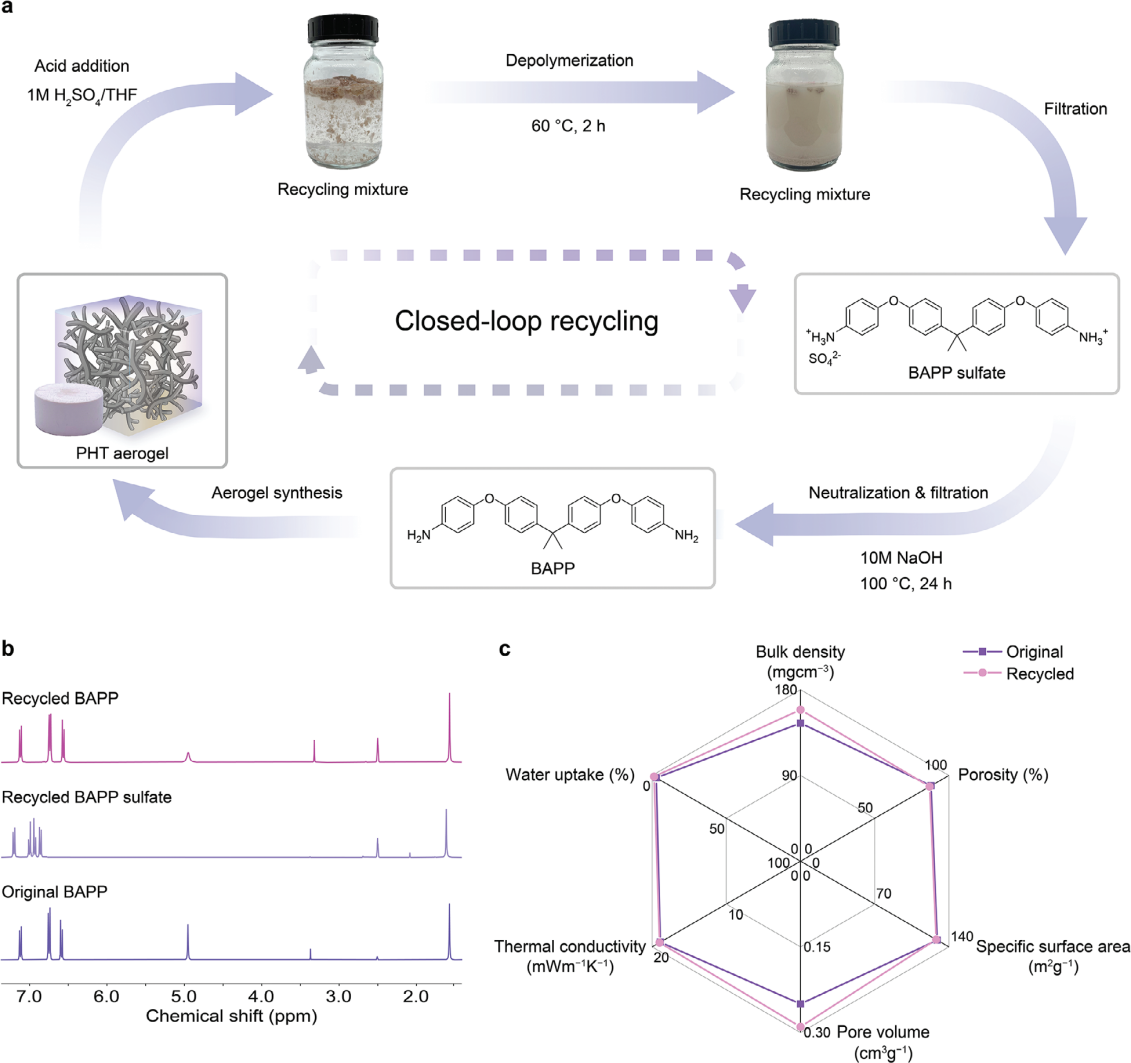
a) Closed‐loop recycling scheme for PHT aerogels. First, PHT aerogels were prepared from BAPP and PFA via aerogel synthesis. Quantitative and selective depolymerization was achieved under acidic conditions at 60 °C (1m H_2_SO_4_/THF, 2/1 w/w) for 2 h. After filtering the BAPP sulfate from the aqueous mixture and drying, it was neutralized under basic conditions at 100 °C (10m NaOH) for 24 h. After filtration and drying, pure BAPP was obtained. b) ^1^H NMR spectra of original BAPP, recycled BAPP sulfate, and recycled BAPP from PHT‐A2. c) Radial graph comparing the aerogel‐specific properties of original PHT‐A2 and recycled PHT‐A2.

PHT aerogels are expected to enable efficient on‐demand depolymerization back to the monomers, as the incorporated HT moieties are known to hydrolyze when treated with aqueous acidic conditions (**Figure** **6**a).^[^ ^42^, ^43^ ^]^ To initiate the chemical closed‐loop recycling of PHT aerogels, we placed samples of PHT‐A2 into an aqueous 1m H_2_SO_4_ solution combined with THF (2/1 w/w) at 60 °C for 2 h. Under these conditions, the aerogel network completely broke down and gave rise to the monomers formaldehyde and BAPP. The liberated formaldehyde was released as a gas but can be easily recovered through distillation when the reaction is performed on an industrial scale.^[^ ^47^, ^48^ ^]^ Meanwhile, the BAPP monomer precipitated as the sulfate salt from the aqueous mixture and was conveniently isolated through simple filtration. The BAPP sulfate was characterized using NMR spectroscopy, and the absence of a signal for methylene groups at a chemical shift of 4.8 ppm validated the complete depolymerization of PHT aerogels. To neutralize the BAPP sulfate, the salt was placed in a 10m aqueous NaOH solution at 100 °C overnight. This process yielded neutral BAPP upon filtration with a recovery yield of 89% and a purity greater than 99%, as confirmed by ¹H NMR spectroscopy (Figure 6b; Figure S9, Supporting Information).

Since the isolated and virgin‐like BAPP monomer was ready for reuse, we prepared a new generation of PHT aerogels (recycled PHT‐A2) using the recycled BAPP together with fresh PFA. Table S4 (Supporting Information) summarizes all the aerogel‐specific properties of recycled PHT‐A2, which are nearly identical to those of the original PHT‐A2 material with low bulk density (160 mgcm^−3^), high porosity (87%), large specific surface area (128 m^2^g^−1^), and intrinsic hydrophobicity (Figure 6c; Figure S10a, Supporting Information). More importantly, the recycled PHT‐A2 achieves a similarly low thermal conductivity of 19 mWm^−1^K^−1^, demonstrating excellent reproducibility of its thermally superinsulating performance. Regarding the microstructural architecture, the recycled aerogel exhibits identical nitrogen physisorption isotherm compared to the one of PHT‐A2 (Figure S10b,c, Supporting Information). Their similar mesoporous structures and overall morphology can also be determined by SEM, where both aerogels show branch‐like scaffolds (Figure S10d, Supporting Information). The thermal and mechanical performance of recycled PHT‐A2 is also in line with the original one, as indicated with similar TGA and stress‐deformation curves (Figure S9e,f, Supporting Information). Overall, we validated the closed‐loop recyclability of our PHT aerogels by successfully fabricating fresh aerogels from recycled monomers.

## 3 Conclusion

Despite their potential to contribute to a sustainable future, chemically recyclable organic aerogels are still underrepresented in the literature.^[^ ^24^, ^25^, ^49^, ^50^, ^51^ ^]^ Comprehensive molecular designs are often involved in creating organic aerogels with high‐performance properties. Our recyclable PHT aerogels demonstrated in this study offer straightforward synthetic access while attaining excellent material properties without additional modification, thereby significantly advancing the field of sustainable thermally insulating materials. The direct use of commercially available feedstocks to prepare PHT aerogels, without any additional modification, enhances their accessibility and practicality for diverse industrial and technological applications. Besides their ultralow thermal conductivity (<20 mWm^−1^K^−1^), low density (<0.2 gcm^−3^), and high thermal stability (*T*_d5%_ > 300 °C), this type of material also exhibits intrinsic hydrophobicity and great mechanical performance. Furthermore, we have shown the efficient recyclability of PHT aerogels by preparing fresh organic aerogel using recycled monomers. While we have explored only a small set of readily available amine precursors, the potential of PHT aerogels is vast and extends far beyond the scope presented here. These materials can be effectively applied to a wide range of other selected precursors, offering significant possibilities for the advancement of circular aerogel, a field that is becoming increasingly important for energy and resource conservation.

## 4 Experimental Section

The experimental details have been provided in the Supporting Information.

## Conflict of Interest

A patent has been filed for this work by the Eindhoven University of Technology

## Supporting information



Supporting Information

## Data Availability

The data that support the findings of this study are available from the corresponding author upon reasonable request.
