# Supplementary material for: One‐Step Synthesis of Closed‐Loop Recyclable and Thermally Superinsulating Polyhexahydrotriazine Aerogels
Source: Adv Mater. 2024 Nov 4;37(1):2412502. doi: 10.1002/adma.202412502 (PMC11707557; doi:10.1002/adma.202412502)
Supplement: Supplementary file 1 — Supporting Information [file ADMA-37-2412502-s001.docx]

**Supporting information**

**One-step synthesis of closed-loop recyclable and thermally superinsulating polyhexahydrotriazine aerogels**

Chang-lin Wang,^[a]^ Yi-Ru Chen, ^[a]^ Fabian Eisenreich,^[a]^ and Željko Tomović*^[a]^

^[a]^ C. Wang, Y.-R. Chen, Dr. F. Eisenreich, Prof. Dr. Ž. Tomović

Polymer Performance Materials Group, Department of Chemical Engineering and Chemistry and Institute for Complex Molecular Systems (ICMS), Eindhoven University of Technology, 5600 MB Eindhoven, The Netherlands.

E-mail: z.tomovic@tue.nl

Table of Contents

**Materials** 3

**Instrumentation** 3

**Model reaction** 5

**Kinetic study and synthesis of hexahydrotriazine (HT)** 5

**Model reactions of HT synthesis using *p-*anisidine and different molar ratios of paraformaldehyde** 6

**Polyhexahydrotriazine (PHT) aerogel synthesis** 6

**Supercritical CO_2_ drying setup** 8

**Closed-loop recycling of PHT aerogels** 9

**Recycled PHT aerogel synthesis** 9

**Results and Discussion** 10

**PHT aerogels fabrication using different monomers and solvents** 10

**Investigation of PHT aerogels prepared from different molar ratio of paraformaldehyde and ODA** 11

**Investigation of mechanical performance of PHT aerogels after prolonged heat exposure** 11

**Discussion on hydrophobicity of PHT aerogels** 11

**Supplementary tables** 12

**Supplementary figures** 15

# **Materials**

Reagent-grade paraformaldehyde (PFA), 4,4'-methylenedianiline (MDA), 4,4'-oxydianiline (ODA), sodium hydroxide (NaOH), and sulfuric acid (>98%) were purchased from Merck life science B.V. *p*-Anisidine, 2,2-bis[4-(4-aminophenoxy)phenyl]propane (BAPP), 9,9-bis(4-aminophenyl)fluorene (FDA), 4,4',4''-triaminotriphenylamine (TAPA), and α,α'-bis(4-aminophenyl)-1,4-diisopropylbenzene (BAPDB) were purchased from BLD Pharmatech Ltd. Ethanol absolute (EtOH), tetrahydrofuran (THF), *N,N*-dimethylformamide (DMF), *N,N*-dimethylacetamide (DMAc), and dimethylsulfoxide (DMSO) were purchased from Biosolve B.V. *N*-methylpyrrolidone (NMP) was purchased from Vivochem B.V. Dimethylsulfoxide-*d*_6_ (99.9% D) and CDCl_3_ (99.9% D) were purchased from Cambridge Isotope Laboratories, Inc. Liquid CO_2_ dip tube grade 2.7, nitrogen grade 5.0, and helium grade 4.6 were purchased by Linde Gas Nederland B.V.

# **Instrumentation**

Nuclear magnetic resonance (NMR) Spectroscopy

The chemical structures of all chemicals were identified by nuclear magnetic resonance using a Bruker UltraShield spectrometer (400 MHz for ^1^H NMR and 100 MHz for ^13^C NMR) at 25 °C using DMSO-*d_6_* or CDCl_3_ as solvent.

Magic angle spinning nuclear magnetic resonance (MAS NMR) spectroscopy

Solid state MAS NMR (Magic Angle Spinning Nuclear Magnetic Resonance) spectra were measured using an 11.7 T Bruker NMR spectrometer operating at 125 MHz for ^13^C NMR spectra, respectively. ^13^C MAS NMR experiments were performed using a Bruker triple channel 4 mm MAS probe head spinning at 13 kHz. ^13^C MAS NMR spectra were recorded using a 13C{1H} cross polarization (CP) pulse sequence with a ramped contact pulse of 1 ms and an interscan delay of 3 s. NMR chemical shift calibrations of ^1^H and ^13^C NMR spectra were done using tetramethylsilane (TMS) and solid adamantane, respectively.

Matrix-assisted laser desorption/ionization-time of flight mass spectrometry

(MALDI-TOF MS)

MALDI-TOF MS of the model compound were recorded on autoflex® maX, Bruker-MALDI-TOF Mass Spectrometer equipped with a 355 nm Nd:YAG smartbeam laser. α-Cyano-4-hydrocycinnamic acid (CHCA) and 2-[(2E)-3-(4-*tert*-butylphenyl)-2-methylprop2-enylidene] malononitrile (DCTB) were used as the matrices. The specimen were solubilized in THF with concentration of 2 mg mL^−1^.

Scanning electron microscopy (SEM)

The morphology of PHT aerogels were characterized by SEM (FEI Quanta 200 3D) at acceleration voltage of 10 kV. The aerogel samples were sputtered with gold for 40 s before testing. PHT aerogels with sample dimensions of 60 mm diameter and 5 mm thickness were used.

Nitrogen physisorption porosimetry

The specific surface area and pore size distribution of the aerogels were analyzed by Brunauer–Emmett–Teller (BET) analyzer (TriStar II Plus). Before measurement, the samples were outgassed at 80 °C for 2 h under vacuum condition. Nitrogen grade 5.0 and Helium grade 4.6 were chosen to measure physisorption isotherm. PHT aerogels with sample dimensions of 60 mm diameter and 5 mm thickness were used.

Helium pycnometry

The porosity and skeletal density of the aerogels were measured by gas pycnometer (AccuPyc II 1345) using Helium grade 4.6. 10 data points were taken with 10 equilibrium cycles. PHT aerogels with sample dimensions of 60 mm diameter and 5 mm thickness were used.

Infrared imaging

An infrared camera (Xeneth 2.5 IR) was used to record the thermographic images and the real-time temperatures of the aerogels placed on a hot stage (100 °C) and cold stage (−25 °C)­ for 10 min with external environment at 20 °C and relative humidity of 45−50%. PHT-A2 aerogel with sample dimensions of 25 mm diameter and 10 mm thickness were used.

Thermogravimetric analysis (TGA)

The thermal properties of PHT aerogels were measured by TGA 550 (TA Instruments) under a nitrogen atmosphere at the heating rate of 10 °C min^−1^ from 40 to 793 °C. PHT aerogels with sample dimensions of 60 mm diameter and 5 mm thickness were used

Thermal conductivity test

The thermal conductivity was measured by heat flow meter (Thermtest Inc., HFM-25) at 20 °C and 20−30% humidity according to ASTM C518 international standard. PHT aerogels with sample dimensions of 65 mm diameter and 5 mm thickness were used. Prior to the measurement, the machine was calibrated with EPS 1450E as reference material.

Contact angle test

The hydrophobicity of the aerogels was studied by a contact angle analyzer (Data-Physics OCA30) at relative humidity of 40%. PHT aerogels with sample dimensions of 65 mm diameter and 5 mm thickness were used.

Uniaxial compression test

Uniaxial compression test was conducted by compression testing machine (ZwickRoell Materials Testing Machine, Zwicki Z2.5/TN). PHT aerogels with sample dimensions of 25 mm diameter and 15 mm thickness were used.

# **Model reaction**

## **Kinetic study and synthesis of hexahydrotriazine (HT)**

*p*-Anisidine (0.50 g, 4.06 mmol, 1 eq.) and paraformaldehyde (0.12 g, 4.06 mmol, 1 eq.) were added to a round-bottom flask and the reagents were dissolved in 6.10 mL of NMP. The reaction mixture was stirred at 100 °C under N_2_ atmosphere. The reaction was monitored by ^1^H NMR spectroscopy to determine the conversion in *p*-anisidine at different time points (1 h, 5 h, and 8 h). To prepare the NMR samples, an aliquot was taken out of the reaction medium (ca. 100 μL) at the selected time points and the solvent was removed under vacuum. The residue was dissolved in CDCl_3_ (400 μL) to perform NMR analysis. After 8 h the reaction was complete and the solvent was removed under vacuum. The product HT was obtained as a brown solid without further purification (0.53 g, 1.31 mmol, 97%). ^1^H NMR (400 MHz, 25 °C, CDCl_3_) δ = 7.06–6.96 (m, 2H), 6.83–6.73 (m, 2H), 4.68 (s, 2H), 3.75 (s, 3H) ppm. ^13^C NMR (100 MHz, 25 °C, CDCl_3_) δ = 154.61, 142.76, 121.03, 120.49, 120.20, 114.88, 114.57, 114.43, 83.40, 55.65 ppm. MS (m/z): [M-H]^-^ calc. for C_24_H_26_N_3_O_3_^−^, 404.20; found, 404.14. The obtained NMR result is in agreement with the reported literatures.^[1,2]^

## **Model reactions of HT synthesis using *p-*anisidine and different molar ratios of paraformaldehyde**

*p*-Anisidine (0.50 g, 4.06 mmol, 1 eq.) and PFA (1, 2, or 4 eq.) were added to a round-bottom flask and the mixture was dissolved in 6.10 mL of NMP. The reactions were initiated by heating to 100 °C and the mixtures were stirred under N_2_ for 8 h. After 8 h, the reaction mixtures were allowed to cool down to room temperature overnight. To prepare NMR samples, aliquots were taken out of the reaction medium (100 μL). Solvent and excess paraformaldehyde were removed under vacuum. After the solvent removal, CDCl_3_ (400 μL) was added for NMR analysis.

# **Polyhexahydrotriazine (PHT) aerogel synthesis**

*General procedure*

All the PHT aerogel preparations were followed by the general procedure, unless mentioned otherwise. The PHT organogel was prepared by mixing components A and B. Component A consists of an aromatic amine dissolved in an organic solvent, while component B consists of paraformaldehyde dissolved in an organic solvent. Both components were prepared in a PP vial by dissolving them in NMP at 100 °C. The amount of paraformaldehyde was kept at an equivalent molar ratio to the amine functional groups of the amine precursor. The gelling was initiated by mixing the two components into one vial at 100 °C. The mixture was shaken until a homogeneous solution was obtained. The solution was poured into a PP vial with 26 mm diameter, and was then placed in an oven at 100 °C until gelation was completed. Afterwards, the organogel was sealed and let aging for 24 h under ambient condition. After aging, the organogel was placed in a solvent bath (300 mL) for solvent exchange. The original solvent was washed out by exchanging the solvent twice, 24 h each time, with 0.1M sodium hydroxide, DI water, and ethanol. The ethanol-saturated gel was then transferred to an autoclave, submerged in ethanol, and sealed in a supercritical fluid-extraction autoclave. The pressure was maintained at 100 bar and the temperature was maintained above 60 °C with the constant inflow of CO_2_. The mixture of solvent and CO_2_ was vented out multiple times during the drying process while withstanding the pressure and temperature. The aerogel was then stored in a nitrogen oven at 80 °C for 2 h to ensure complete removal of the solvent. The dried sample was stored in a desiccator chamber with relative humidity of 30% to prevent possible moisture uptake.

The detailed formulation for preparing PHT aerogels is summarized in **Table S1**, **Table S3**, and **Table S5**. Different multifunctional amines, including 2,2-bis[4-(4-aminophenoxy)phenyl]propane (BAPP), 4,4'-methylenedianiline (MDA), 4,4'-oxydianiline (ODA), 9,9-bis(4-aminophenyl)fluorene (FDA), 4,4',4''-triaminotriphenylamine (TAPA), and α,α'-bis(4-aminophenyl)-1,4-diisopropylbenzene (BAPDB) as well as different organic solvents, including *N,N*-dimethylformamide (DMF), *N,N*-dimethylacetamide (DMAc), dimethylsulfoxide (DMSO), and *N*-methylpyrrolidone (NMP) were utilized to prepare PHT aerogels (**Scheme S1**). The initial concentration to prepare the organogels was set to 0.12 g/mL (solute mass/solvent volume,) and the mass of the solvent was kept constant at 9 g. In **Table S1**, the aerogels were labeled based on aromatic amines and organic solvents used. Take PHT-a1 and PHT-b2 as example, ‘a’ stands for BAPP as amine and ‘b’ stands for MDA as amine; ‘1’ stands for NMP as organic solvent and ‘2’stands for DMF as organic solvent. In **Table S1**, the aerogel samples with the names of PHT-a2−4, PHT-b2−4, PHT-c2−4, PHT-d1−4, PHT-e1−4, and PHT-f1−4 were prepared using the general procedure of PHT aerogel synthesis except from their solvent exchange procedures. In this case, the samples after aging were placed in an ethanol bath (100 mL) for solvent exchange for three times with 24 h each times.

In the manuscript, we chose PHT-a1 as a promising candidate, which was prepared using BAPP as the amine and NMP as the organic solvent for future development and improvement. To investigate the correlation between initial amine concentration (in the range of 7 to 13 wt%) and material properties of the final aerogels. The aerogel samples were prepared in different initial concentration and labeled accordingly as PHT-A1 to PHT-A4 throughout the manuscript (**Table S3**). The PHT aerogels were prepared using the general procedure of PHT aerogels. While preparing the organogel, the solution was poured into a PP mold with 70 mm in diameter.

To confirm the chemical structure of hexahydrotriazine (HT) and hemiaminal (HA) observed from model studies, two organic aerogels based on different molar ratios between ODA and PFA were prepared and labeled as PHT-O1 and PHT-O2 (**Table S5**). The PHT-O aerogels were prepared according to the general procedure.


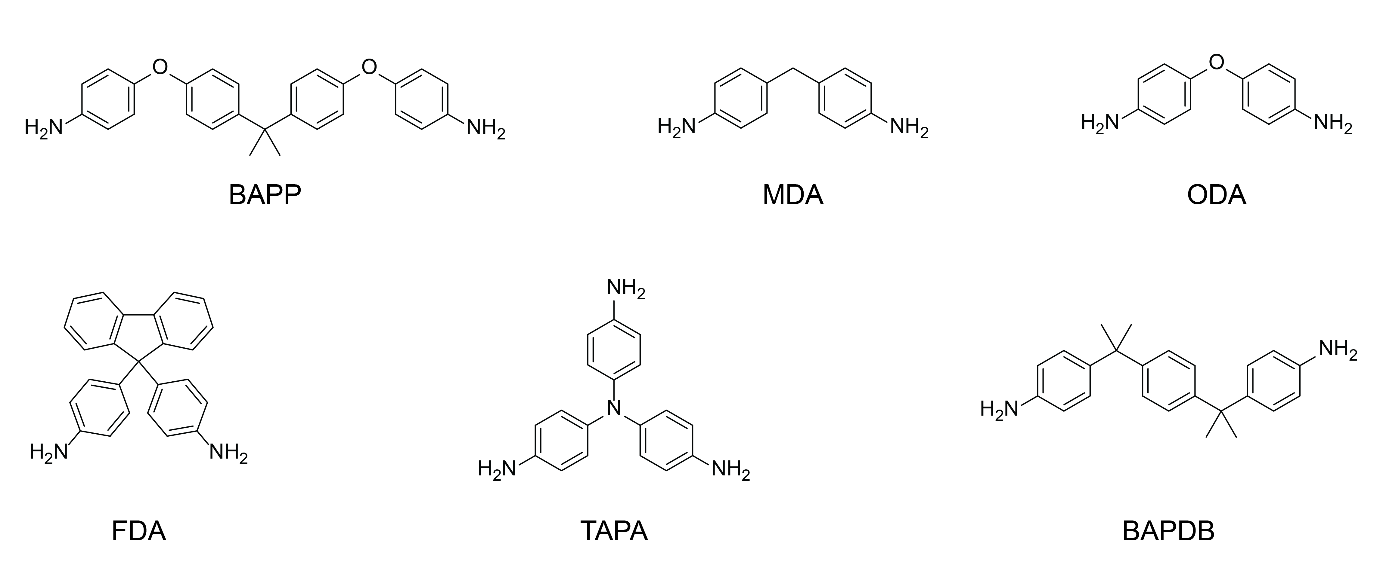


**Scheme S1.** Chemical structures of different multifunctional aromatic amines used in this study.

# **Supercritical CO_2_ drying setup**

Liquid CO_2_ grade 2.7 (purity > 99.7%) is used as exchange agent for the supercritical drying process (SCD). The high pressure extraction/drying units “HP-DE200” is utilized as the drying setup. It comprises one autoclave (**Scheme S2**), provided by Eurotechnica, with a maximum working temperature of 100 °C and allowable operation pressure of 220 bar. The autoclave includes a thermowell with a NiCr-Ni thermocouple to measure the internal temperature during the process. Two venting tubes are also attached to the autoclave to extract the covering solvent and depressurization. Apart from the autoclave (9), the supercritical drying system employed in this work consists of two thermal baths (Selecta, UNITRONIC 200) for heat exchanger 1 and 2 (4 and 7), a mechanical pump (5) (provided by Maximator), a check number valve (2), 5 needle valve (3,6,7,8,10 and 11) and a CO_2_ bottle (1) (**Scheme S2**).

Gels are first introduced into the autoclave (9) and covered with the solvent used for gel formation. This was done to avoid premature solvent evaporation that could lead to a higher shrinkage. Then, CO_2_ is gradually pressurized up to 100 bar, extracting the solvent from the inside of the wet gel pores. The heat exchanger 2 (7) maintains the autoclave at constant temperature of 60 °C. Once supercritical conditions are achieved, the solvent is extracted from the gels in the autoclave (9) (**Scheme S2**). The supercritical CO_2_ enriched with extracted solvent is vented out by releasing the autoclave. During this process, the pressure is maintained above with constant fresh CO_2_ input. The venting process takes around 10 to 15 min and the autoclave will be closed to reach further extraction. Three cycles of extraction were further conducted with waiting interval of 30 min each. Finally, when the aerogel pores are completely free of solvent, pressure is slowly released to atmospheric pressure through the metering valve for 45 min.

**
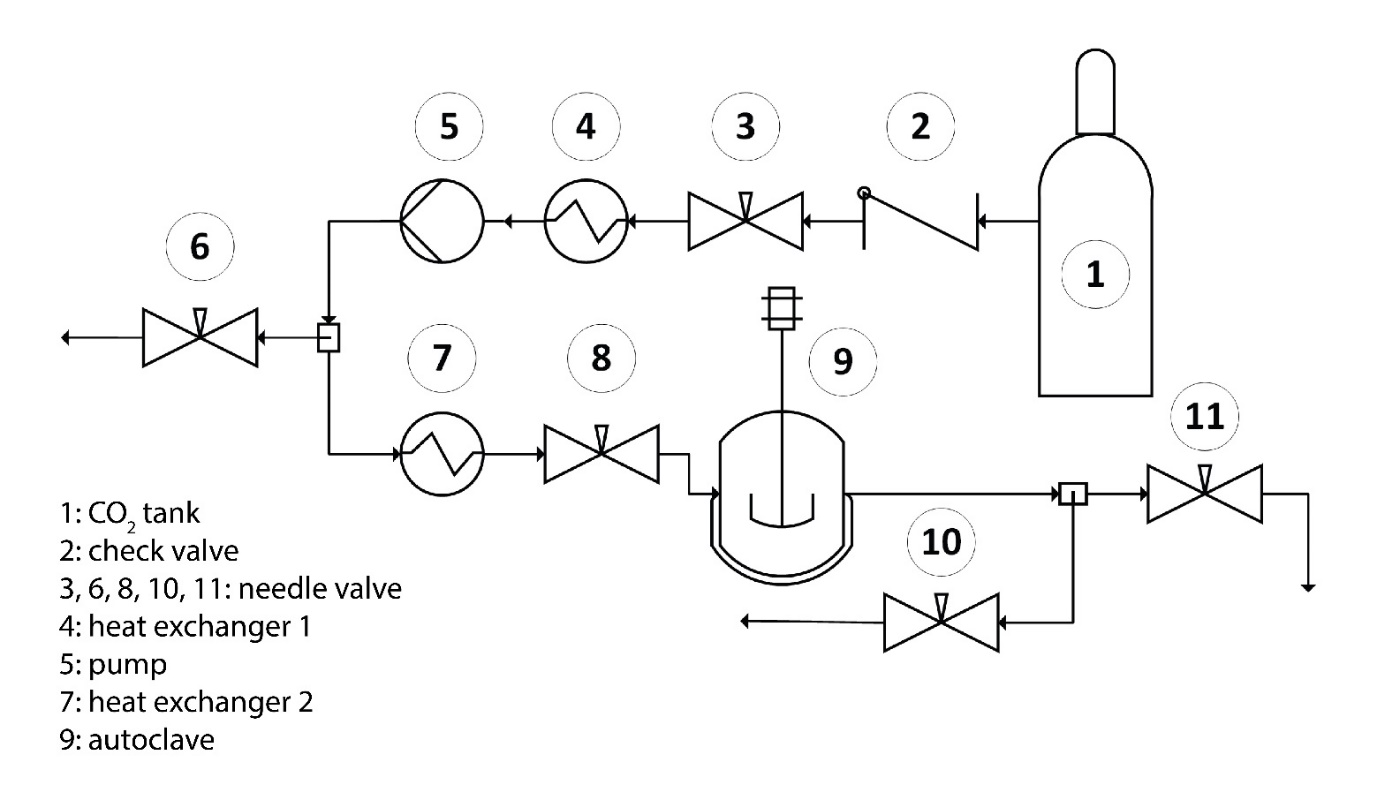
**

**Scheme S2.** Flow diagram of supercritical drying setup.

# **Closed-loop recycling of PHT aerogels**

4 g of PHT-A2 were added to 50 g of 1M H_2_SO_4_ and 25 g of THF in a 150 mL vial (**Table S3**). The mixture was stirred at 60 °C for 2 h. After complete depolymerization, the precipitated BAPP sulfate was filtered, washed with distilled water and acetone, and dried in a vacuum oven at 60 °C for overnight. The BAPP salt was then neutralized with 30 mL of 10M NaOH solution at 100 °C for 24 h. The precipitated BAPP was filtered, washed with distilled water, dried in a vacuum oven at 60 °C overnight, and finally obtained as off-white powders in 89% yield.

# **Recycled PHT aerogel synthesis**

The preparation of recycled PHT aerogels follows the general procedure of the PHT aerogels synthesis, where recycled BAPP was employed. Herein, 2.29 g BAPP dissolved in 11.3 g NMP was mixed with 0.34 g PFA dissolved in 11.2 g NMP to prepare the aerogel with the size of 60 mm in diameter and 5 mm in height.

# **Results and Discussion**

## **PHT aerogels fabrication using different monomers and solvents**

The creation of aerogel structures is significantly influenced by the solvents and starting monomers used in the process. This study aims to investigate the effects of different solvents and monomers on the properties of PHT aerogels. A total of six different multifunctional aromatic amines and four solvents were utilized for the study (**Table S1**). Based on different amines and solvent used, the samples were labeled as PHT-a1 to PHT-f4. The corresponding chemical structures and abbreviations of the aromatic amines used can be found in **Figure S3**. According to **Table S2**, it reveals significant variations in the material properties of PHT aerogels depending on the combination of solvents and monomers utilized. For example, the use of DMF or DMAc as solvents resulted in PHT-a2 and PHT-a3 with microporous structure, while NMP as a gelling medium produced a high specific surface area of up to 132 m^2^g^−1^ (PHT-a1). To prepare highly thermally insulating materials, PHT-a1 was selected for further investigation and will be discussed in detail in the manuscript due to its intricate mesoporous structure. On the other hand, PHT-b1−4, PHT-c1−4, and PHT-f1−4 all exhibited low specific surface areas below 100 m^2^g^−1^, despite their low bulk density and high porosity. This is due to the absence of mesopores within their structures, hindering their potential as thermally superinsulating materials. Additionally, the use of FDA allowed for the creation of PHT aerogels with high specific surface areas and minimal shrinkage (PHT-ds). However, PHT-d1 did not show strong mechanical performance, where the samples show notable brittles after compression (**Figure S11a**). Similarly, PHT-es boasted high specific surface areas and large pore volumes. However, the sample completely absorbed water after submerging in distilled for 24 h, its hydrophilic nature thus made it unsuitable for use as high performance aerogels (**Figure S11b**). Through this study, we have demonstrated the influence of varying solvent and monomer combinations on the properties of PHT aerogels. Our focus on developing thermally superinsulating materials led us to choose PHT-a1 as a promising candidate, which prepared using BAPP as the amine and NMP as the organic solvent for future development and improvement. To investigate the correlation between initial amine concentration (in the range of 7 to 13 wt%) and material properties of the final aerogels. The aerogel samples were prepared in different initial concentration and labeled accordingly as PHT-A1 to PHT-A4 throughout the manuscript (**Table S3**).

## **Investigation of PHT aerogels prepared from different molar ratio of paraformaldehyde and ODA**

To confirm the chemical structure of HT and HA observed from model studies, two organic aerogels based on different molar ratios between ODA and PFA were prepared, namely PHT-O1 and PHT-O2 (**Table S5**). The synthesis of the PHT-O follows the general procedure of PHT aerogel synthesis. After the fabrication of PHT-O aerogels, ^13^C MAS NMR experiments were carried out to investigate the potential presence of HT. As shown in **Figure S3**, all spectra are presented with the methylene moiety from HT at 70 ppm. The chemical shift observed at 90 ppm of PHT-O2 is originated from the CH_2_-OH groups connected to the HA structure, confirming the incomplete trimerization of aromatic amines. To further investigate the impact of the HA on thermal performance, we performed TGA analysis. As shown in **Figure S12**, PHT-O2, which contains HA, exhibited poor thermal performance, with a decomposition temperature (*T*_d5%_) at 5% weight loss occurring at 99 °C. In contrast, PHT-O1, consisting solely of PHT, showed excellent thermal performance with *T*_d5%_ around 300 °C. It indicates that HA containing PHT aerogels show poor thermal performance.

## **Investigation of mechanical performance of PHT aerogels after prolonged heat exposure**

To investigate the mechanical stability of PHT aerogels upon heat exposure, we performed uniaxial compression testing of PHT-A2 after heat treatment to assess any changes. After the preparation of PHT-A2, the samples were subjected to heat treatment in an air oven at 120 °C for varying durations of 0 h, 3 h, 10 h, and 24 h. Compression testing was performed directly after the heat treatment. Based on the stress-deformation curves, the specimens demonstrated similar mechanical performance across all conditions (**Figure S13**). Therefore, we can conclude that prolonged exposure to elevated temperatures does not significantly affect the mechanical properties of PHT aerogels.

## **Discussion on hydrophobicity of PHT aerogels**

The hydrophobicity of organic aerogels can be influenced by various factors, such as polymer characteristics, porosity, and surface roughness.^[3,4]^ A recent study on the PHT polymer indicated that PHT does not exhibit strong water repellence, with water contact angle values ranging from 73° to 81°.^[5]^ Hence, it suggests that the mesoporous morphology of our PHT aerogels, prepared from sol-gel synthesis, grants these materials with intrinsic hydrophobic characteristics.

# **Supplementary tables**

**Table S1. Formulation of PHT aerogels using different solvents and monomers**

| Name^a)^ | Solvent  type | BAPP  [g] | MDA  [g] | ODA  [g] | FDA  [g] | TAPA  [g] | BAPDB  [g] | PFA  [g] | Solvent mass  [g] |
| --- | --- | --- | --- | --- | --- | --- | --- | --- | --- |
| PHT-a1 | NMP | 0.92 | - | - | - | - | - | 0.13 | 9 |
| PHT-a2 | DMF | 1.00 | - | - | - | - | - | 0.15 | 9 |
| PHT-a3 | DMAc | 1.00 | - | - | - | - | - | 0.15 | 9 |
| PHT-a4 | DMSO | 0.86 | - | - | - | - | - | 0.13 | 9 |
| PHT-b1 | NMP | - | 0.81 | - | - | - | - | 0.24 | 9 |
| PHT-b2 | DMF | - | 0.88 | - | - | - | - | 0.26 | 9 |
| PHT-b3 | DMAc | - | 0.88 | - | - | - | - | 0.27 | 9 |
| PHT-b4 | DMSO | - | 0.75 | - | - | - | - | 0.23 | 9 |
| PHT-c1 | NMP | - | - | 0.81 | - | - | - | 0.24 | 9 |
| PHT-c2 | DMF | - | - | 0.88 | - | - | - | 0.27 | 9 |
| PHT-c3 | DMAc | - | - | 0.88 | - | - | - | 0.27 | 9 |
| PHT-c4 | DMSO | - | - | 0.76 | - | - | - | 0.23 | 9 |
| PHT-d1 | NMP | - | - | - | 0.89 | - | - | 0.15 | 9 |
| PHT-d2 | DMF | - | - | - | 0.98 | - | - | 0.17 | 9 |
| PHT-d3 | DMAc | - | - | - | 0.98 | - | - | 0.17 | 9 |
| PHT-d4 | DMSO | - | - | - | 0.84 | - | - | 0.14 | 9 |
| PHT-e1 | NMP | - | - | - | - | 0.80 | - | 0.25 | 9 |
| PHT-e2 | DMF | - | - | - | - | 0.87 | - | 0.27 | 9 |
| PHT-e3 | DMAc | - | - | - | - | 0.88 | - | 0.27 | 9 |
| PHT-e4 | DMSO | - | - | - | - | 0.75 | - | 0.23 | 9 |
| PHT-f1 | NMP | - | - | - | - | - | 0.89 | 0.16 | 9 |
| PHT-f2 | DMF | - | - | - | - | - | 0.97 | 0.17 | 9 |
| PHT-f3 | DMAc | - | - | - | - | - | 0.98 | 0.17 | 9 |
| PHT-f4 | DMSO | - | - | - | - | - | 0.84 | 0.15 | 9 |

^a)^ All the aerogel are made in the size of 25 mm in diameter

**Table S2. Material properties of PHT aerogels using different solvents and monomers**

| Name^a)^ | Bulk density  *ρ_b_*  [mgcm^−3^] | Linear shrinkage  [%] | Skeletal density  *ρ_s_*  [gcm^−3^] | Porosity  Π  [%] | Specific surface area  [m^2^g^−1^] | Pore volume [cm^3^g^−1^] |
| --- | --- | --- | --- | --- | --- | --- |
| PHT-a1 | 166 | 17 | 1.23 | 87 | 132 | 0.23 |
| PHT-a2 | 136 | 12 | 1.25 | 89 | Macropore^b)^ | Macropore^b)^ |
| PHT-a3 | 139 | 11 | 1.25 | 89 | Macropore^b)^ | Macropore^b)^ |
| PHT-a4 | X ^c)^ | X | X | X | X | X |
| PHT-b1 | 143 | 11 | 1.22 | 88 | 27.8 | 0.06 |
| PHT-b2 | 170 | 16 | 1.22 | 86 | 68.4 | 0.22 |
| PHT-b3 | 146 | 14 | 1.22 | 87 | 35.3 | 0.08 |
| PHT-b4 | 169 | 16 | 1.22 | 86 | 63.1 | 0.16 |
| PHT-c1 | 147 | 13 | 1.33 | 89 | 56.5 | 0.16 |
| PHT-c2 | 143 | 15 | 1.26 | 89 | 71.4 | 0.23 |
| PHT-c3 | 157 | 15 | 1.26 | 88 | 22.1 | 0.09 |
| PHT-c4 | 157 | 13 | 1.31 | 88 | 74.1 | 0.37 |
| PHT-d1 | 157 | 6 | 1.30 | 90 | 536.7 | 0.63 |
| PHT-d2 | 152 | 6 | 1.23 | 88 | 348.7 | 0.66 |
| PHT-d3 | 114 | 5 | 1.24 | 91 | 321.5 | 0.52 |
| PHT-d4 | 112 | 5 | 1.24 | 91 | 317.7 | 0.50 |
| PHT-e1 | 142 | 10 | 1.38 | 90 | 310.9 | 0.52 |
| PHT-e2 | 129 | 8 | 1.22 | 89 | 283.4 | 0.74 |
| PHT-e3 | 147 | 11 | 1.42 | 90 | 573.3 | 1.68 |
| PHT-e4 | 156 | 13 | 1.23 | 87 | 485.5 | 1.12 |
| PHT-f1 | 150 | 10 | 1.15 | 91 | 32.6 | 0.07 |
| PHT-f2 | 129 | 12 | 1.11 | 88 | 91.0 | 0.18 |
| PHT-f3 | 155 | 7 | 1.11 | 86 | 77.1 | 0.31 |
| PHT-f4 | 119 | 7 | 1.12 | 89 | 16.3 | 0.06 |

^a)^The sample with the size of 25 mm in diameter were used ^b)^No nitrogen adsorption can be detected. ^c)^It is not possible to obtain a stable organogel.

**Table S3. Formulation of PHT aerogels**

| Name | BAPP  [g] | PFA  [g] | NMP  [g] |
| --- | --- | --- | --- |
| PHT-A1 | 1.72 | 0.25 | 22.5 |
| PHT-A2 | 2.29 | 0.34 | 22.5 |
| PHT-A3 | 2.86 | 0.42 | 22.5 |
| PHT-A4 | 3.43 | 0.50 | 22.5 |
| PHT-A1^a)^ | 0.69 | 0.10 | 9 |
| PHT-A2^a)^ | 0.92 | 0.13 | 9 |
| PHT-A3^a)^ | 1.14 | 0.17 | 9 |
| PHT-A4^a)^ | 1.37 | 0.20 | 9 |

^a)^The samples were prepared for compression testing with sample dimensions of 25 mm diameter and 15 mm thickness were used.

**Table S4. Material properties of Recycled PHT aerogel**

| **Recycled PHT-A2** | **Value** |
| --- | --- |
| Bulk density *ρ_b_* [mgcm^−3^] | 160 |
| Linear shrinkage [%]^a)^ | 11 |
| Skeletal density *ρ_s_* [gcm^−3^] | 1.19 |
| Porosity Π [%]^b)^ | 87 |
| Specific surface area [m^2^g^−1^] | 127.7 |
| Pore volume [cm^3^g^−1^] | 0.29 |
| Skeleton width [nm] | 36.9±8 |
| Contact angle [°] | 144±3 |
| Water uptake [%] | 1.30 |
| Young’s modulus [MPa] | 1.24 |
| Compressive strength [MPa]^c)^ | 0.46 |
| Thermal conductivity [mWm^−1^K^−1^] | 19.0 |
| Decomposition temperature at 5% weight loss *T*_d5%_ [°C] | 349 |
| Char yield at 793 °C [%] | 13.7 |

^a)^Linear shrinkage was calculated based on the diameter change of the sample; ^b)^Porosity was calculated via equation: *Π*=(1-ρ_b_/ρ_s_ )×100%; ^c)^Compressive strength at 10% deformation ratio.

**Table S5. Formulation of PHT aerogels using ODA**

| Name | ODA  [g] | PFA  [g] | NMP  [g] |
| --- | --- | --- | --- |
| PHT-O1 | 0.92 | 0.13 | 9 |
| PHT-O2 | 0.48 | 0.57 | 9 |

# **Supplementary figures**


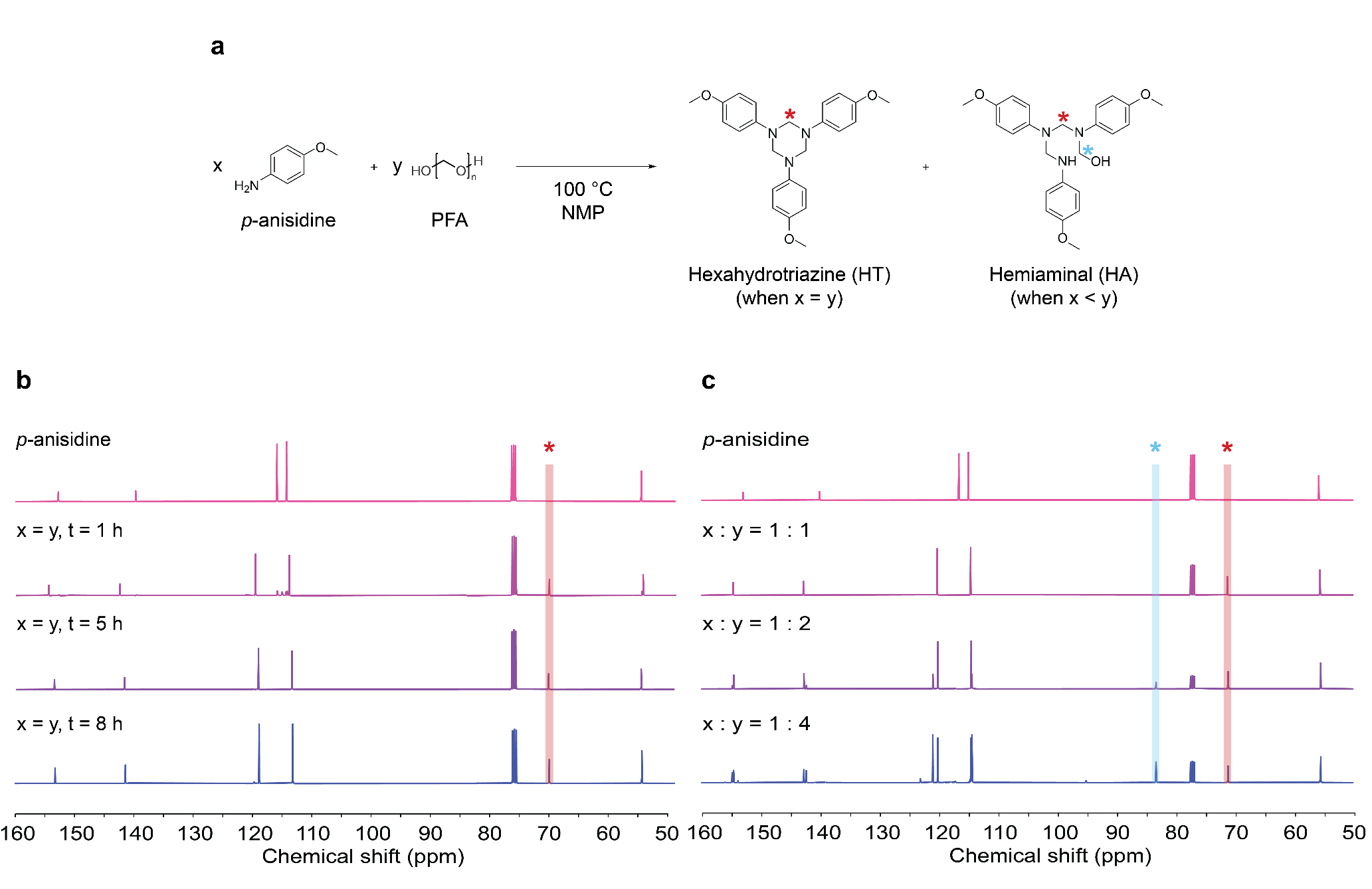


**Figure S1.** a) ^13^C NMR spectra (100 MHz, 25 °C, CDCl_3_) of the reaction between *p*-anisidine and equivalent ratio of paraformaldehyde. b) ^13^C NMR spectra (100 MHz, 25 °C, CDCl_3_) of the reaction between *p*-anisidine and different molar ratio of paraformaldehyde (1, 2, and 4) after 8 h.


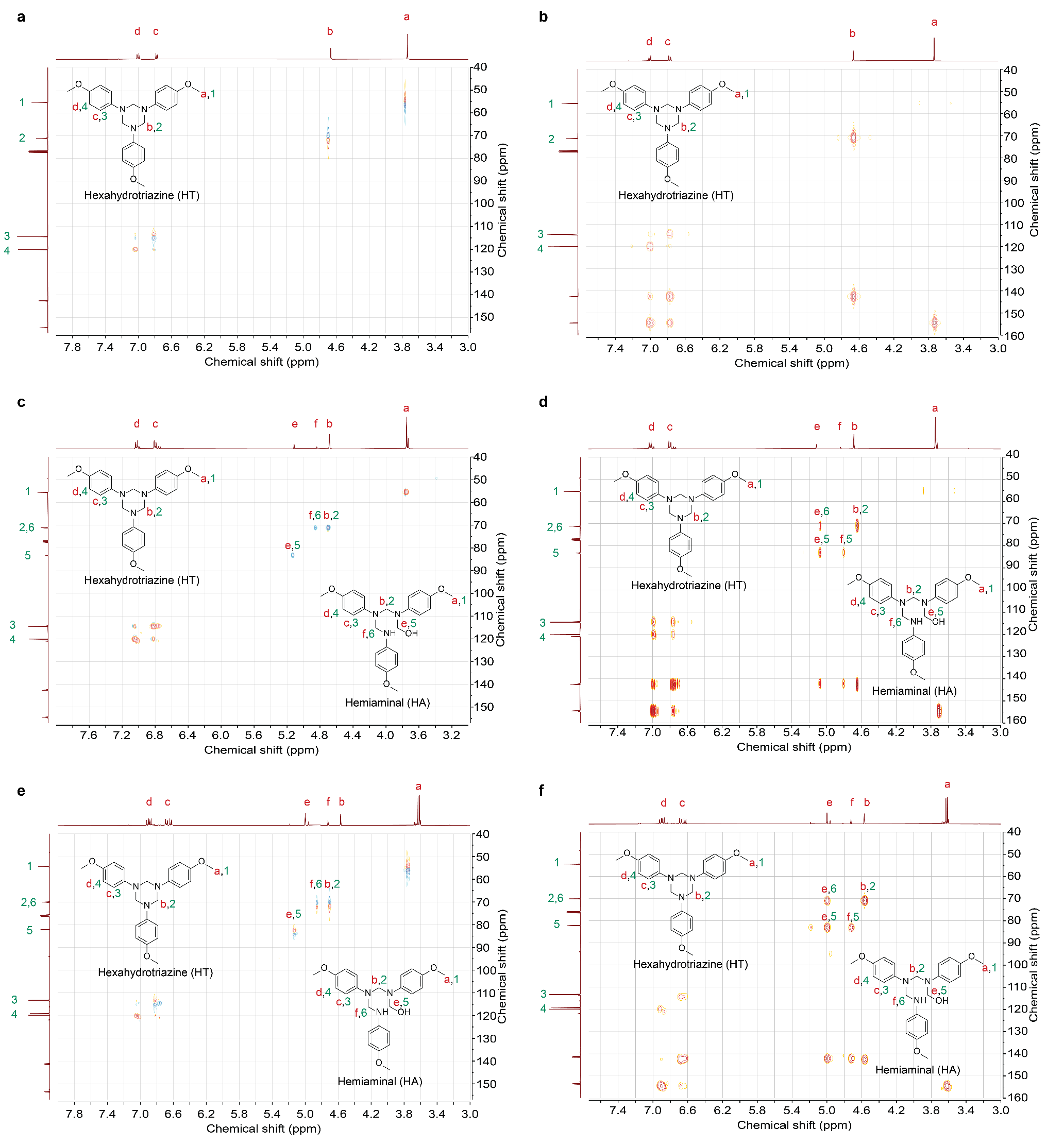


**Figure S2.** 2D NMR spectroscopy of the model compounds of hexahydrotriazine (HT) and hemiaminal (HA) prepared from *p*-anisidine and different molar ratios of paraformaldehyde. a) ^1^H-^13^C HSQC spectra and b) ^1^H-^13^C HMBC spectra of HT prepared from 1 to 1 molar equivalents of *p*-anisidine and paraformaldehyde; c) ^1^H-^13^C HSQC spectra and d) ^1^H-^13^C HMBC spectra of HT and HA prepared from 1 to 2 molar equivalents of *p*-anisidine and paraformaldehyde; e) ^1^H-^13^C HSQC spectra and f) ^1^H-^13^C HMBC spectra of HT and HA prepared from 1 to 4 molar equivalents of *p*-anisidine and paraformaldehyde.


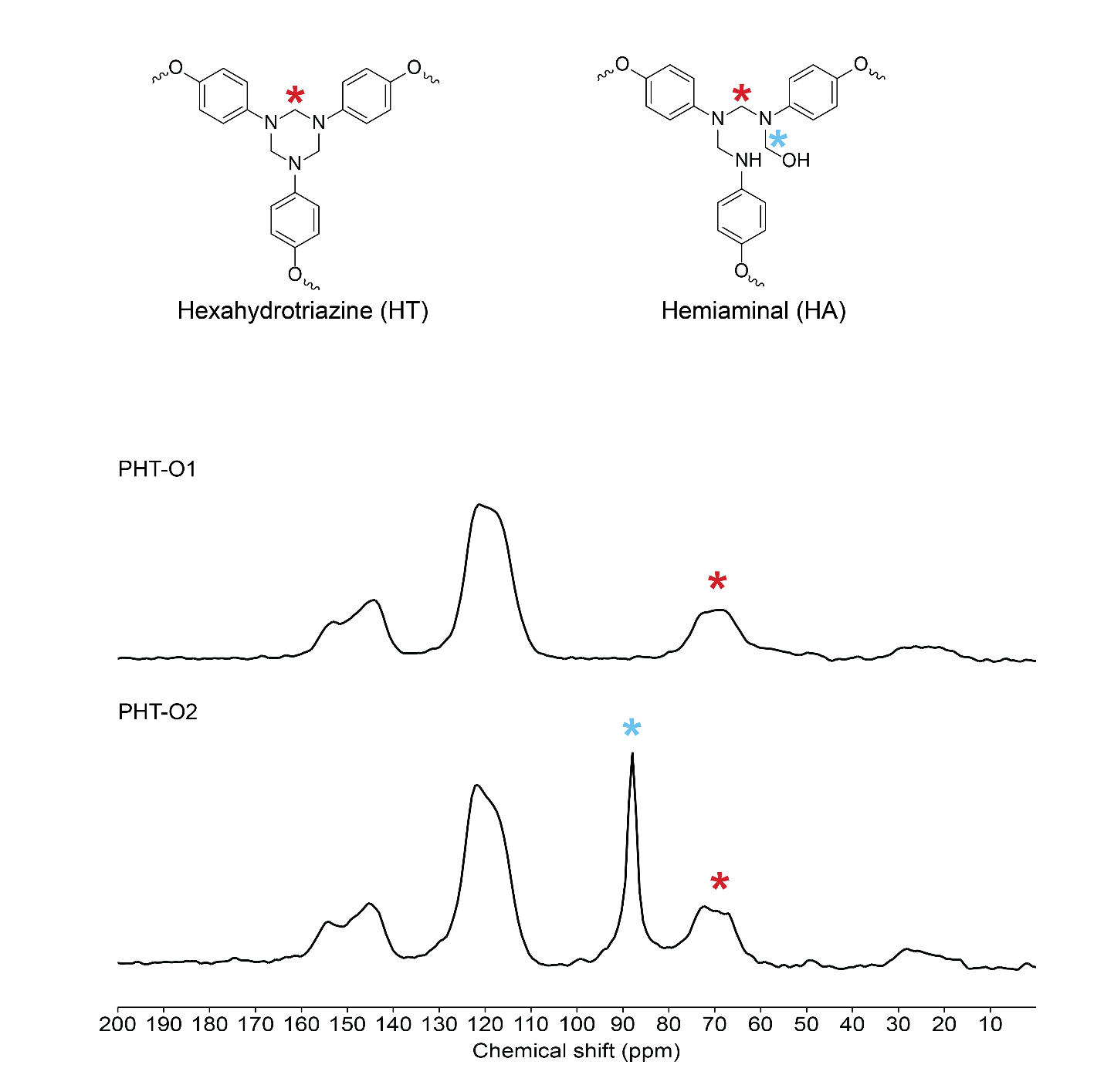


**Figure S3.** Solid-state ^13^C MAS NMR spectra of PHT-O1 and PHT-O2. PHT-O1 is prepared from 1 to 1 molar equivalent of amine groups from ODA and paraformaldehyde; PHT-O2 is prepared from 1 to 4 molar equivalent of amine groups from ODA and paraformaldehyde.


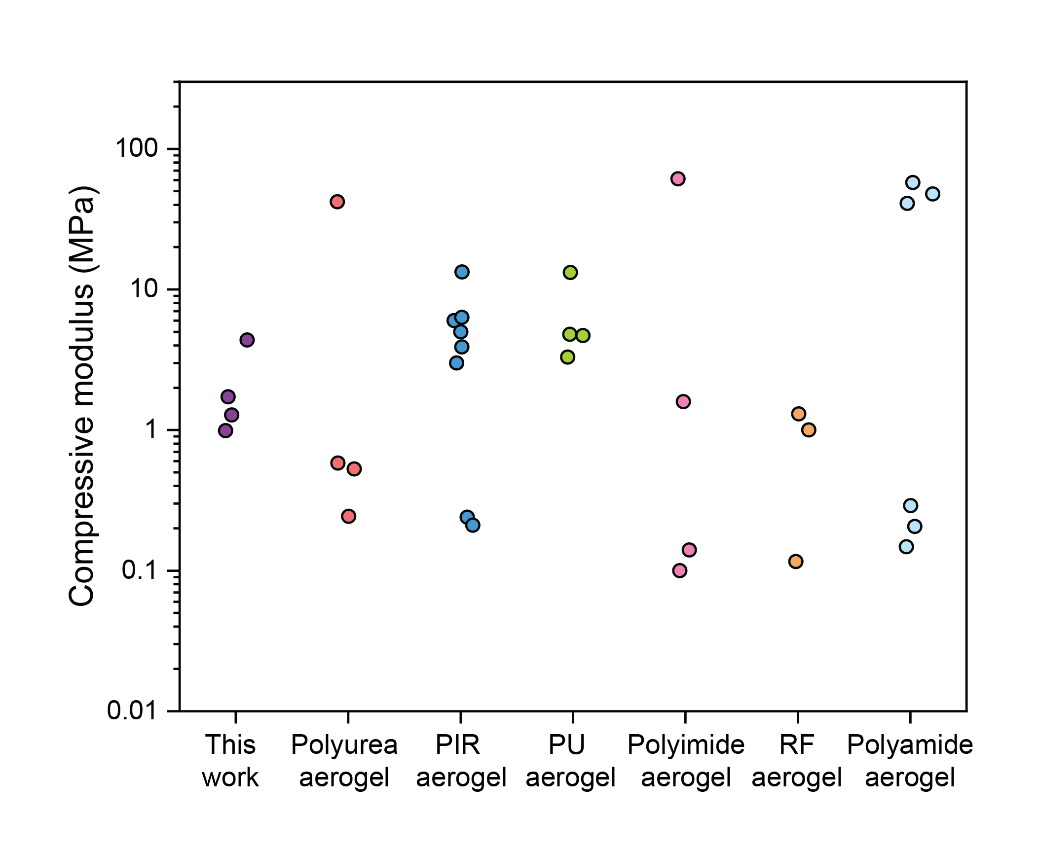


**Figure S4.** Comparison of compressive modulus of PHT aerogels with other organic aerogels, including polyurea aerogels^[6,7]^, polyisocyanurate (PIR) aerogels^[4,8,9]^, polyurethane (PU) aerogels^[10,11]^, polyimide aerogels^[12–15]^, resorcinol-formaldehyde (RF) aerogels^[16,17]^, and polyamide aerogels^[18,19]^.


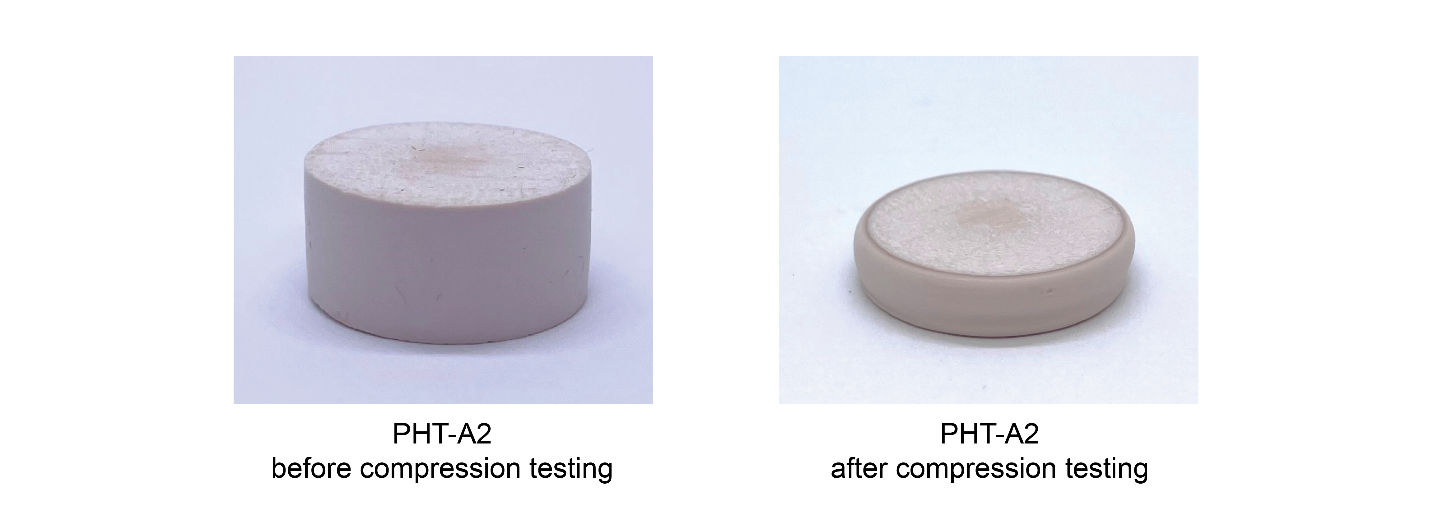


**Figure S5.** Photograph of PHT-A2 before and after the uniaxial compression testing.


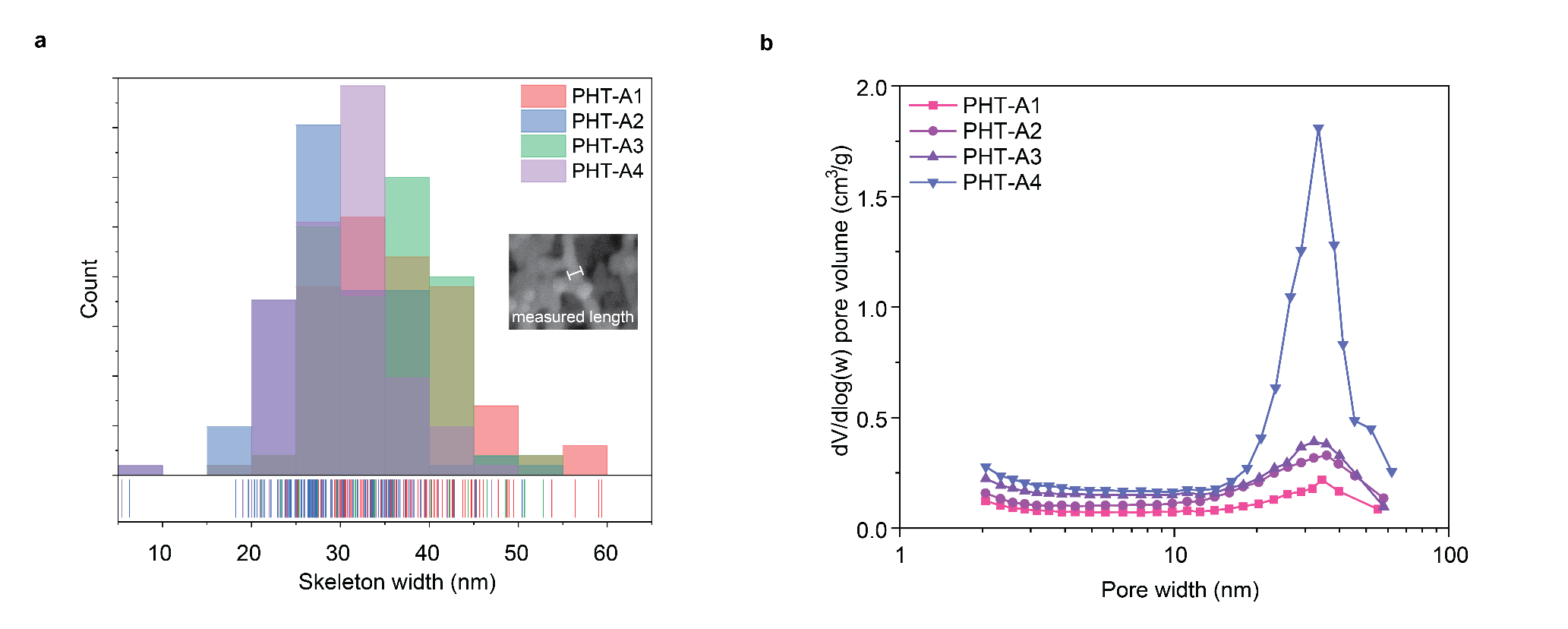


**Figure S6.** a) Histogram of skeleton width distribution of PHT aerogels. b) Pore size distribution of PHT aerogels based on N_2_ porosimetry.


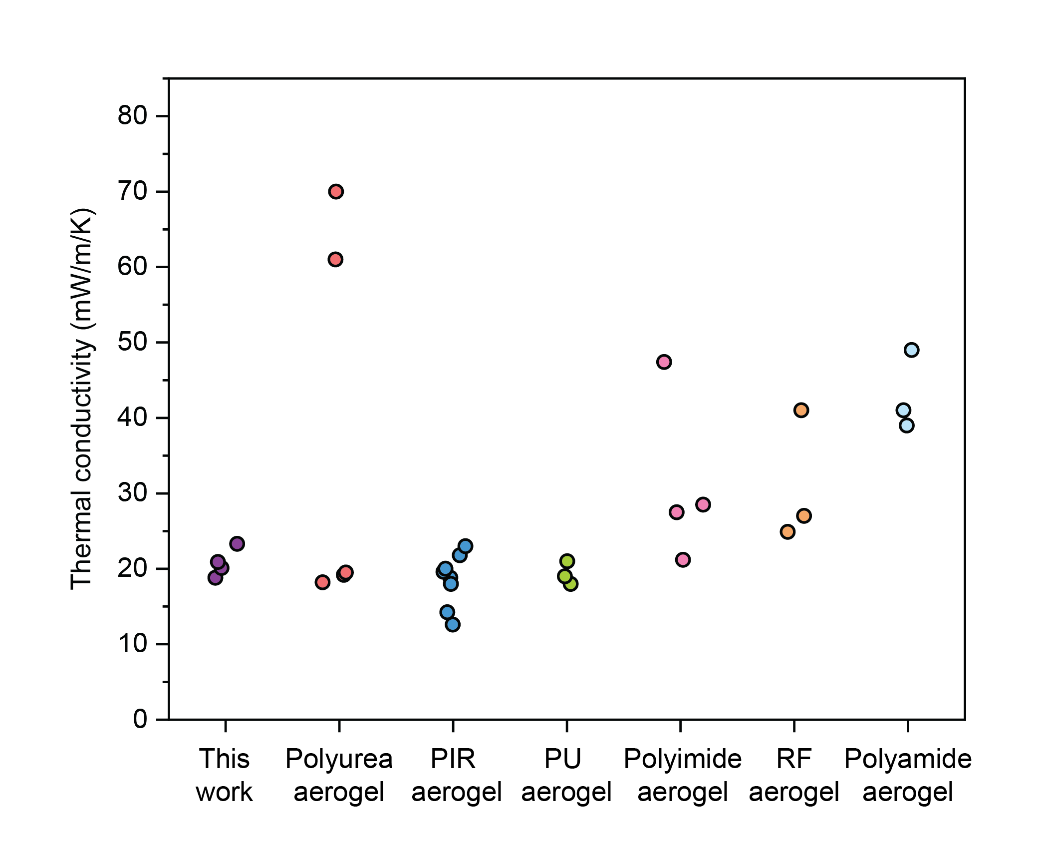


**Figure S7.** Comparison of thermal conductivity of PHT aerogels with other organic aerogels, including polyurea aerogels^[6,7]^, polyisocyanurate (PIR) aerogels^[4,8,9]^, polyurethane (PU) aerogels^[10,11]^, polyimide aerogels^[12–15]^, resorcinol-formaldehyde (RF) aerogels^[16,17]^, and polyamide aerogels^[18,19]^.


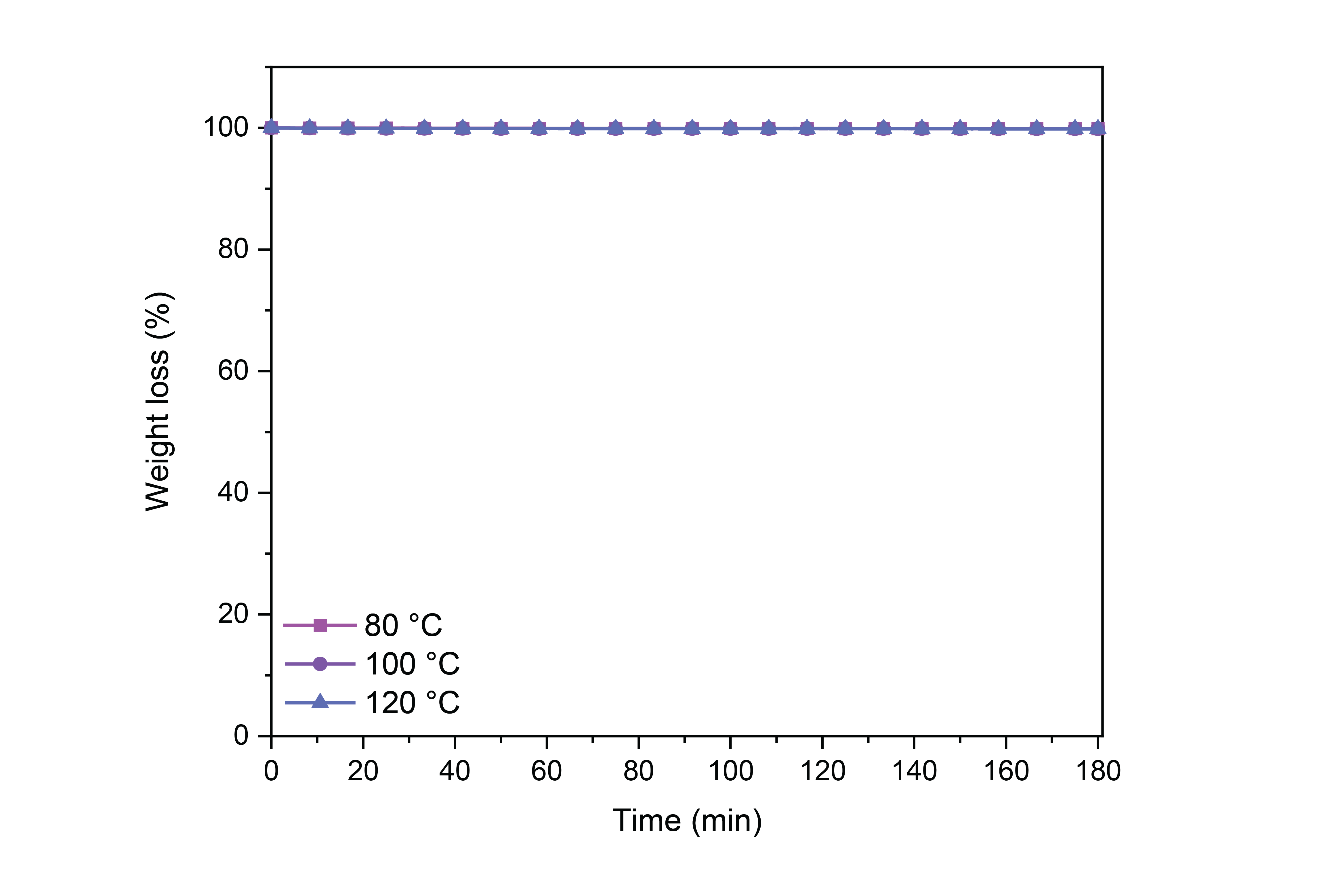


**Figure S8.** TGA curves of PHT-A2 demonstrating its thermal stability at different temperatures, that is 80 °C, 100 °C, and 120 °C, over time.


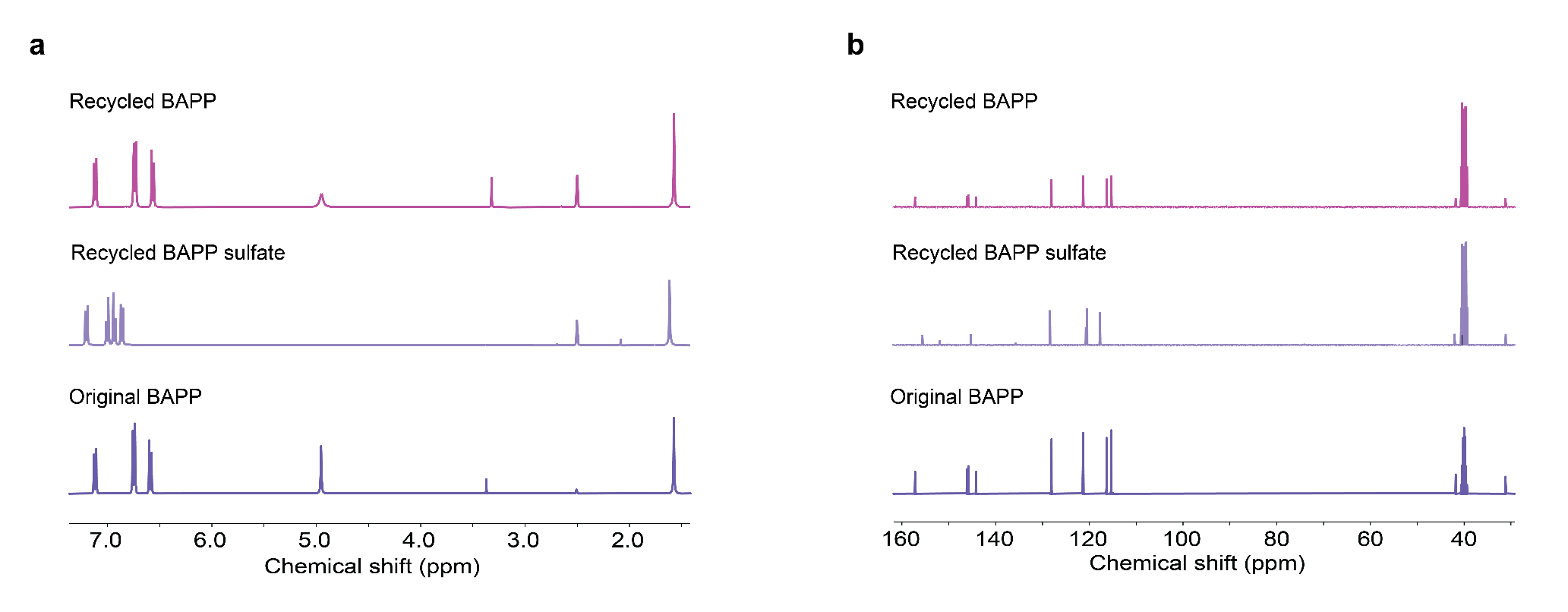


**Figure S9.** a) ^1^H NMR (400 MHz, 25 °C) and b) ^13^C NMR (100 MHz, 25 °C) spectra of original BAPP, recycled BAPP sulfate and recycled BAPP in DMSO-*d*_6_ as solvent.

**
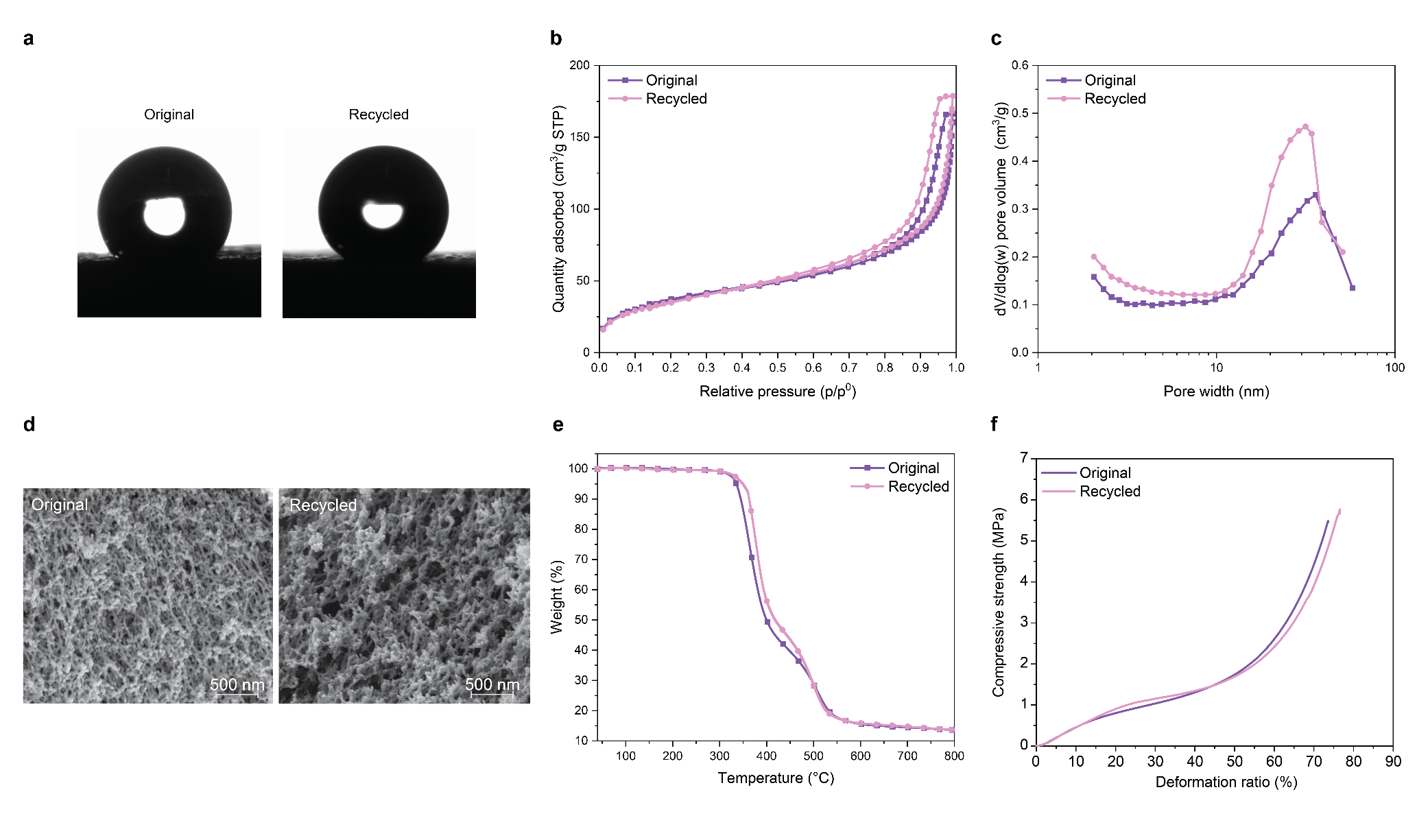
**

**Figure S10**. a) Water contact angle images of original and recycled PHT aerogels. b) Nitrogen physisorption isotherms of original and recycled PHT aerogels. c) Pore size distribution of original and recycled PHT aerogels based on N_2_ porosimetry. d) SEM images of original and recycled PHT aerogels. e) TGA curves of PHT aerogels ramping from 40 to 793 °C with ramp rate of 10 °C/min. f) Compression-deformation curves of original and recycled PHT aerogels.


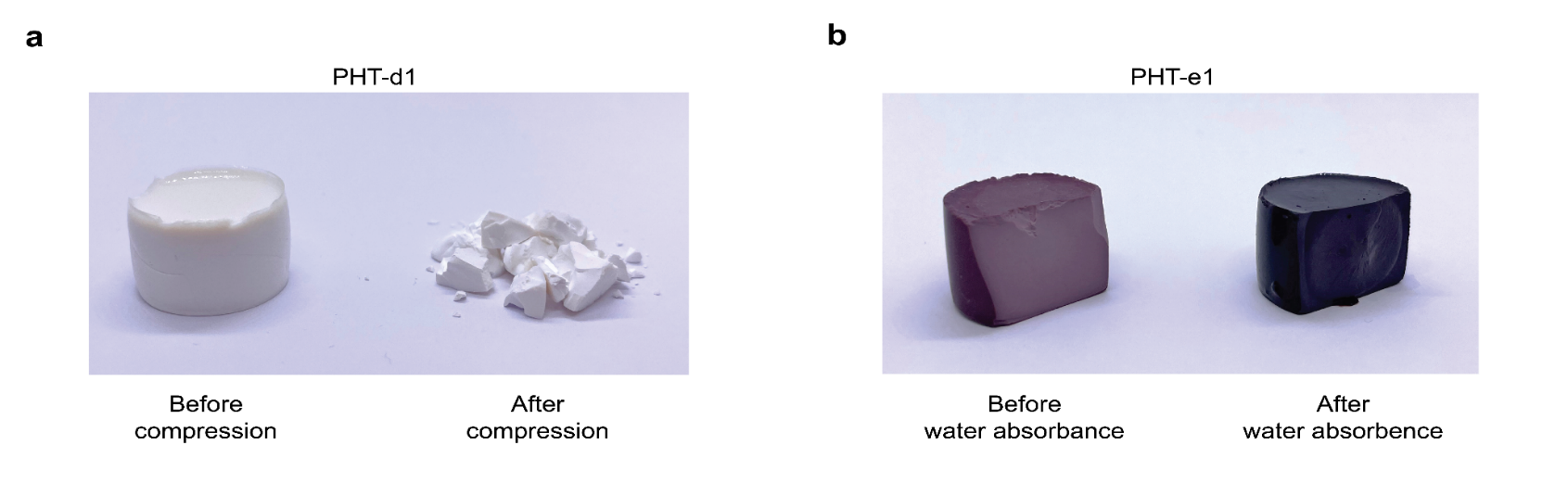


**Figure S11**. a) Photograph of PHT-d1 before and after compression. b) Photograph of PHT-e1 before and after water absorbance. The samples were cut after the testing in order to show the water absorbance effect.

*
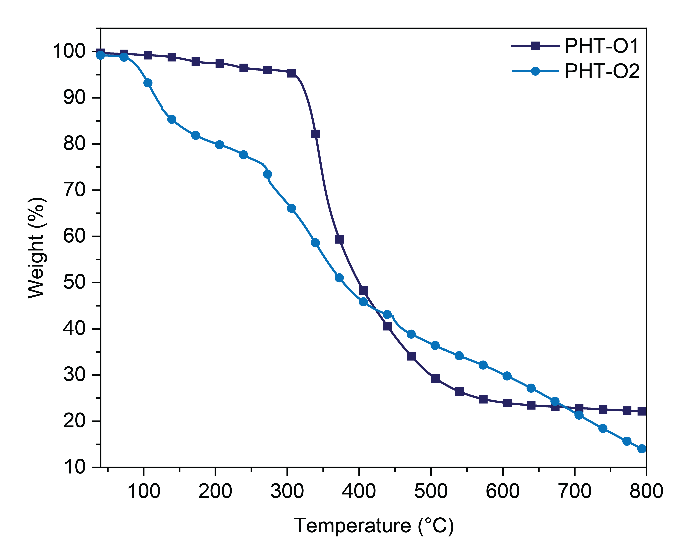
*

**Figure S12.** TGA curves of PHT-O1 and PHT-O2 ramping from 40 to 793 °C with ramp rate of 10 °C/min.


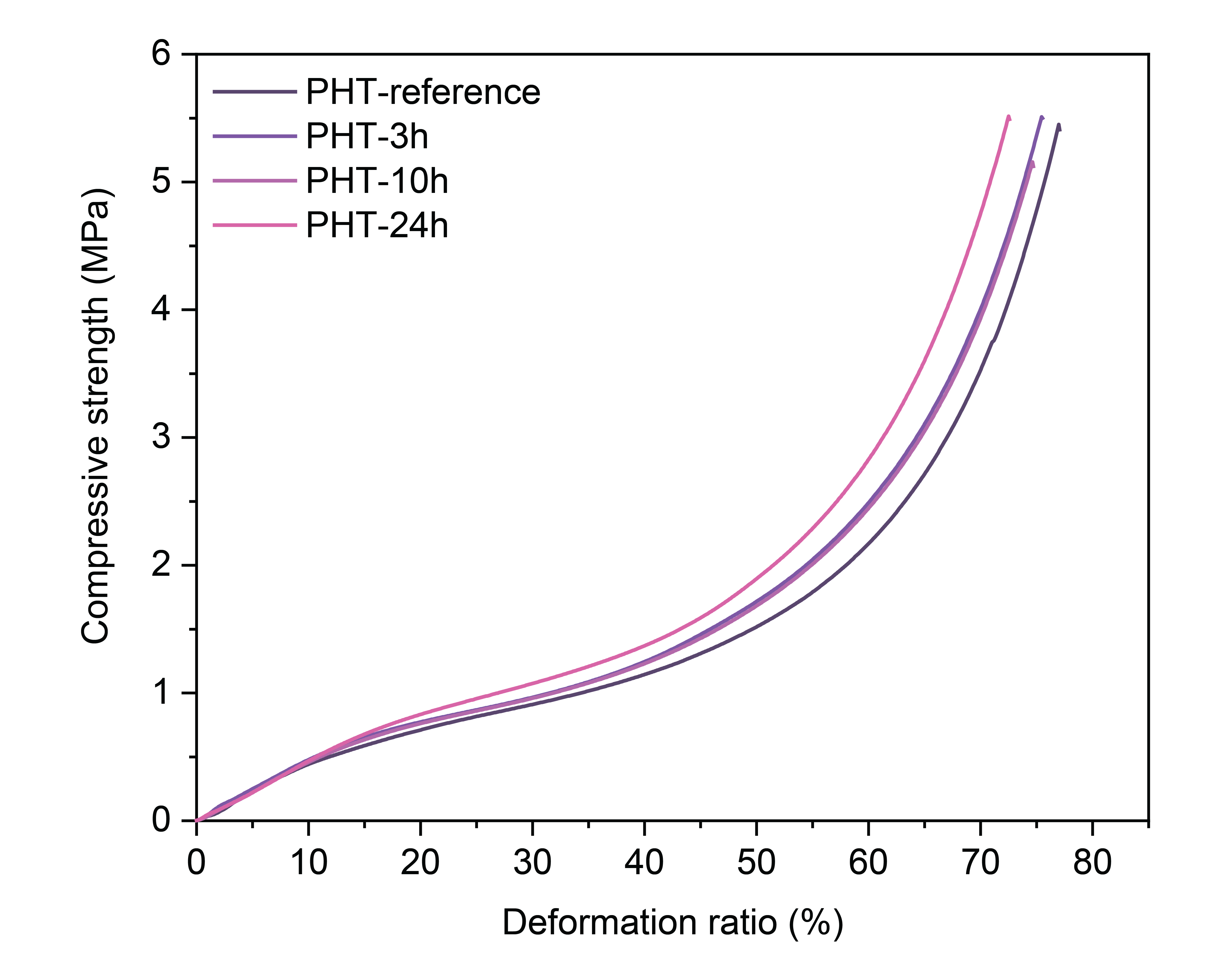


**Figure S13.** Stress-deformation curves of PHT-A2 after placing in an air oven at 120 °C for different time durations: PHT-reference (0 h), PHT-3h (3 h), PHT-10h (10 h), and PHT-24h (24 h.)

**References**

[1] G. Bondle, R. Jadhav, V. Kamble, S. Atkore, *Lett. Org. Chem.* **2017**, *14*, 18.

[2] A. Martínez-Manjarres, R. Quevedo, *J. Mol. Struct.* **2021**, *1236*, 22.

[3] S. Donthula, C. Mandal, T. Leventis, J. Schisler, A. M. Saeed, C. Sotiriou-Leventis, N. Leventis, *Chem. Mater.* **2017**, *29*, 4461.

[4] T. Taghvaee, S. Donthula, P. M. Rewatkar, H. Majedi Far, C. Sotiriou-Leventis, N. Leventis, *ACS Nano* **2019**, *13*, 3677.

[5] C. Min, Y. Yang, H. Liang, Z. He, Q. Li, J. Zhu, K. Zhang, H. Shi, W. Wang, *ACS Appl. Polym. Mater.* **2021**, *3*, 5257.

[6] J. K. Lee, G. L. Gould, W. Rhine, *J. Sol-Gel Sci. Technol.* **2009**, *49*, 209.

[7] A. M. Saeed, C. A. Wisner, S. Donthula, H. Majedi Far, C. Sotiriou-Leventis, N. Leventis, *Chem. Mater.* **2016**, *28*, 4867.

[8] N. Leventis, C. Chidambareswarapattar, A. Bang, C. Sotiriou-Leventis, *ACS Appl. Mater. Interfaces* **2014**, *6*, 6872.

[9] N. Diascorn, S. Calas, H. Sallée, P. Achard, A. Rigacci, *J. Supercrit. Fluids* **2015**, *106*, 76.

[10] R. Trifu, G. Gould, S. White, *MRS Adv.* **2017**, *325*, 1.

[11] B. Merillas, F. Villafañe, M. Á. Rodríguez-Pérez, *Nanomaterials* **2022**, *12*, DOI 10.3390/nano12142409.

[12] J. Tian, Y. Yang, T. Xue, G. Chao, W. Fan, T. Liu, *J. Mater. Sci. Technol.* **2022**, *105*, 194.

[13] Z. Ma, T. Xue, Q. Wali, Y. E. Miao, W. Fan, T. Liu, *Compos. Commun.* **2023**, *39*, 101528.

[14] O. A. Tafreshi, S. Ghaffari-Mosanenzadeh, S. Karamikamkar, Z. Saadatnia, S. Kiddell, C. B. Park, H. E. Naguib, *J. Mater. Chem. C* **2022**, *10*, 5088.

[15] K. Yao, S. Jiang, S. Li, C. Zhang, H. Hou, *Compos. Commun.* **2023**, *38*, 101503.

[16] M. Alshrah, M. P. Tran, P. Gong, H. E. Naguib, C. B. Park, *J. Colloid Interface Sci.* **2017**, *485*, 65.

[17] M. Schwan, R. Tannert, L. Ratke, *J. Supercrit. Fluids* **2016**, *107*, 201.

[18] H. Ren, J. Zhu, Y. Bi, Y. Xu, L. Zhang, *J. Porous Mater.* **2017**, *24*, 1165.

[19] J. C. Williams, M. A. B. Meador, L. McCorkle, C. Mueller, N. Wilmoth, *Chem. Mater.* **2014**, *26*, 4163.
